# A Pathologically Friendly Strategy for Determining the Organ‐specific Spatial Tumor Microenvironment Topology in Lung Adenocarcinoma Through the Integration of snRandom‐seq and Imaging Mass Cytometry

**DOI:** 10.1002/advs.202308892

**Published:** 2024-04-29

**Authors:** Xuqi Sun, Xiao Teng, Chuan Liu, Weihong Tian, Jinlin Cheng, Shuqiang Hao, Yuzhi Jin, Libing Hong, Yongqiang Zheng, Xiaomeng Dai, Linying Wu, Lulu Liu, Xiaodong Teng, Yi Shi, Peng Zhao, Weijia Fang, Yu Shi, Xuanwen Bao

**Affiliations:** ^1^ Department of Medical Oncology The First Affiliated Hospital Zhejiang University School of Medicine Hangzhou 310003 China; ^2^ Department of Thoracic Surgery The First Affiliated Hospital Zhejiang University School of Medicine Hangzhou 310003 China; ^3^ Changzhou Third People's Hospital Changzhou Medical Center Nanjing Medical University 140 Hanzhong Rd, Gulou Nanjing Jiangsu 210029 China; ^4^ State Key Laboratory for Diagnosis and Treatment of Infectious Diseases The First Affiliated Hospital Zhejiang University School of Medicine Hangzhou 310003 China; ^5^ State Key Laboratory of Oncology in South China Collaborative Innovation Center for Cancer Medicine Sun Yat‐sen University Cancer Center Guangzhou 510060 China; ^6^ Department of Respiratory Disease The First Affiliated Hospital College of Medicine Zhejiang University Hangzhou 310003 China; ^7^ Department of Pathology The First Affiliated Hospital Zhejiang University School of Medicine Hangzhou 310003 China; ^8^ Bio‐X Institutes Key Laboratory for the Genetics of Developmental and Neuropsychiatric Disorders Shanghai Jiao Tong University 1954 Huashan Road Shanghai 200030 China

**Keywords:** lung adenocarcinoma, organ‐specific landscapes, single‐cell nuclei RNA sequencing, spatial topology, tumor microenvironments

## Abstract

Heterogeneous organ‐specific responses to immunotherapy exist in lung cancer. Dissecting tumor microenvironment (TME) can provide new insights into the mechanisms of divergent responses, the process of which remains poor, partly due to the challenges associated with single‐cell profiling using formalin‐fixed paraffin‐embedded (FFPE) materials. In this study, single‐cell nuclei RNA sequencing and imaging mass cytometry (IMC) are used to dissect organ‐specific cellular and spatial TME based on FFPE samples from paired primary lung adenocarcinoma (LUAD) and metastases. Single‐cell analyses of 84 294 cells from sequencing and 250 600 cells from IMC reveal divergent organ‐specific immune niches. For sites of LUAD responding well to immunotherapy, including primary LUAD and adrenal gland metastases, a significant enrichment of B, plasma, and T cells is detected. Spatially resolved maps reveal cellular neighborhoods recapitulating functional units of the tumor ecosystem and the spatial proximity of B and CD4^+^ T cells at immunogenic sites. Various organ‐specific densities of tertiary lymphoid structures are observed. Immunosuppressive sites, including brain and liver metastases, are deposited with collagen I, and T cells at these sites highly express TIM‐3. This study originally deciphers the single‐cell landscape of the organ‐specific TME at both cellular and spatial levels for LUAD, indicating the necessity for organ‐specific treatment approaches.

## Graphical abstract

The mechanism for organ‐specific responses of lung cancer to immunotherapy remains unclear. By using both snRNA‐sequencing and imaging mass cytometry, this study deciphered the heterogeneous single‐cell landscape of organ‐specific tumor microenvironment at both cellular and spatial levels in lung cancer patients and provided potential treatment paradigms for metastases resistant to immunotherapy.

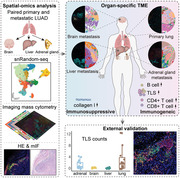

## 1 Introduction

Lung cancer is the leading cause of cancer‐related mortality worldwide, with lung adenocarcinoma (LUAD) accounting for approximately 40% of the cases.^[^ ^1^ ^]^ LUAD is frequently diagnosed at an advanced stage with extrathoracic metastases such as in the brain and liver.^[^ ^2^ ^]^ Despite curative resection, patients with early‐stage LUAD still suffer from distant metastases, most of which occur within two years of surgery.^[^ ^3^ ^]^ The five‐year overall survival rate of patients with metastatic non‐small cell lung cancer (NSCLC) remains approxiamtely 20%.^[^ ^4^, ^5^, ^6^ ^]^ In the past decade, immune checkpoint inhibitors (ICIs), represented by programmed death‐1 (PD‐1) blockades, have achieved promising efficacy in metastatic NSCLC patients. However, the objective response rate is less than 50%.^[^ ^7^, ^8^, ^9^ ^]^ Subgroup analyses revealed divergent responses of different metastatic organs to PD‐1 blockades.^[^ ^10^, ^11^, ^12^ ^]^ Specifically, PD‐1 blockade is less effective in liver and brain metastases than in metastases at other anatomical sites, such as lymph nodes and adrenal glands.^[^ ^13^, ^14^ ^]^ The poor response leads to dismal survival, which indicates the unmet need to understand the potential mechanism of organ‐specific responses to optimize treatments for metastatic lesions resistant to immunotherapy.

The tumor microenvironment (TME) is a highly structured ecosystem composed of tumor cells, immune cells, stromal cells, and extracellular matrix.^[^ ^15^ ^]^ Emerging evidence has revealed that the TME thoroughly regulates the efficiency of immunotherapy based on bulk RNA sequencing (RNA‐seq) or single‐cell RNA‐sequencing (scRNA‐seq).^[^ ^16^, ^17^, ^18^ ^]^ By integrating NSCLC single‐cell datasets, Salcher et al. uncovered that tissue‐resident neutrophils contributed to the failure of PD‐L1 blockade treatment.^[^ ^19^ ^]^ Moreover, NSCLC patients with different responses to PD‐1 blockade have distinct TME remodeling processes during immunotherapy. Increased precursor‐exhausted T cells and decreased aged CCL3^+^ neutrophils were observed in responsive tumors, but these trends were not detected in refractory ones.^[^ ^20^, ^21^ ^]^ The positioning of immune cells can also dictate their functions.^[^ ^22^ ^]^ Andrew et al. found that NSCLC patients with more baseline cellular modules consisting of PDCD1^+^ CXCL13^+^ activated T cells, SPP1^+^ macrophages, and IgG^+^ plasma cells have better responses to PD‐(L)1 blockades.^[^ ^23^ ^]^

Previous scRNA‐seq studies have mainly focused on the heterogeneity of primary lesions, whereas organ‐specific immunity against metastases has barely been explored. The multitudinous interactions between cells and the extracellular matrix in the TME shape the organ‐specific milieu, which deeply influences tumor response to immunotherapy.^[^ ^24^ ^]^ Different response patterns can be observed when classified by metastatic sites, even within the same patient cohort.^[^ ^11^, ^25^, ^26^ ^]^ However, current clinical guidelines hardly consider organ‐specific treatment approaches due to the lack of a comprehensive understanding of organ‐specific tumor ecosystems based on high‐dimensional data. Although a few studies have recently explored the TME of LUAD metastases using scRNA‐seq, they have only delineated the TME of brain metastases.^[^ ^27^, ^28^ ^]^ In addition, primary and metastatic lesions were not paired in these studies, leading to potential inter‐patient and inter‐tumoral heterogeneity. To date, the organ‐specific TME of LUAD has not been comprehensively elucidated, either at the cellular or molecular level or in terms of spatial characteristics.

Depicting the organ‐specific TME of LUAD at single‐cell resolution is logistically and technically challenging due to the lack of paired treatment‐naïve surgical specimens for primary and metastatic lesions. Systemic therapy is the standard first‐line treatment for patients with synchronous metastases and multi‐site tissue biopsies are not routinely performed. Obtaining paired primary tumors and metachronous metastases can be particularly challenging, as many patients receive treatments at different hospitals and the surgical criteria for patients with metachronous metastasis are extremely stringent. In this context, formalin‐fixed paraffin‐embedded (FFPE) tissue sections are the optimal modality for sample collection; however, they are not suitable for conventional scRNA‐seq. Recently, a droplet‐based high‐throughput single‐nuclei RNA sequencing method has been developed that can capture full‐length total RNAs with random primers (snRandom‐seq) from single cells in FFPE tissue sections.^[^ ^29^ ^]^ Furthermore, scRNA‐seq cannot reveal spatial topology owing to tissue dissociation, a limitation addressed by imaging mass cytometry (IMC), which enables spatial phenotyping of the TME.

In this study, we dissected the cellular and spatial heterogeneity of the organ‐specific TME in paired primary LUAD and metastases in different organs at single‐cell resolution, using snRandom‐seq and IMC. The results unveiled the potential mechanism for divergent responses to immunotherapy across tumor lesions at different anatomical sites and paved the way for organ‐specific treatment paradigms.

## 2 Results

### 2.1 Single‐Cell Landscape of Paired Primary LUAD and Metastases in Different Organs

**Figure 1:**
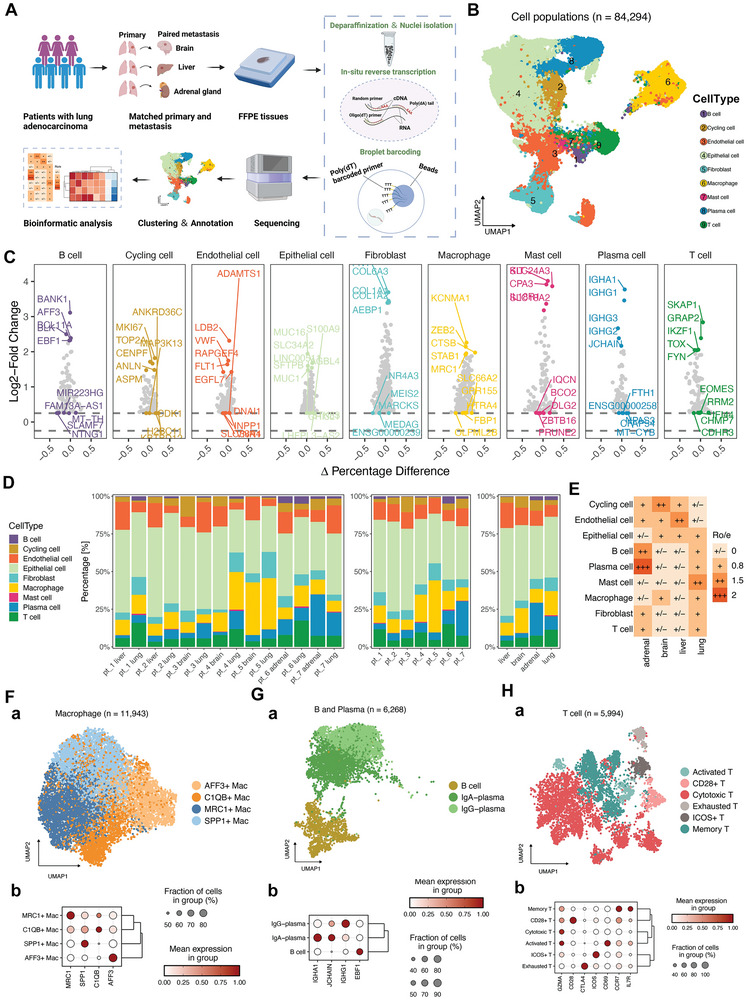
High‐resolution tumor microenvironment atlas of paired primary LUAD and metastases in the brain, liver, and adrenal gland depicted by scRandom‐seq. A) Overview of the design for snRandom‐seq. FFPE samples from paired primary and metastatic lesions were collected for single‐cell nuclei transcriptomic sequencing. B) UMAP plot of major cell types from all the samples. C) The mean expressions of canonical marker genes for the major clusters. D) The frequency of each cell cluster presented as a proportion of total cells in each sample. E) Tissue prevalence of each cell cluster estimated by the Ro/e analysis. F–H) UMAP plots of macrophages, B and plasma cells, and T cells show distinct subclusters. Dot plots of expression levels of classical markers for each subclusters.

To characterize the organ‐specific TME of LUAD at single‐cell resolution, we performed snRandom‐seq on 14 treatment‐naïve FFPE tissue samples from paired primary LUAD (*n =* 7) and metastases to the brain (*n =* 3), liver (*n =* 2), and adrenal gland (*n =* 2) (**Figure** **1**A). The unsupervised clustering analysis classified the 84294 cells into nine clusters annotated with canonical markers (Figure 1B). Each cluster was identified as a broad cell type: B cells (1480 cells, marked with EBF1, BCL11A, and BANK1); cycling cells (4079 cells, marked with MKI67 and TOP2A); endothelial cells (8585 cells, marked with EGFL7, VWF, ADAMTS1, and FLT1); epithelial cells (37314 cells, marked with MUC1, MUC 16 and SFTPB); fibroblasts (5965 cells, marked with FN1, COL1A1‐2 and COL6A3); macrophages (11943 cells, marked with CTSB, SPP1, and MRC1); mast cells (330 cells, marked with CAP3 and KIT); plasma cells (7246 cells, marked with IGHA1, IGHG1‐3, and JCHAIN); and T cells (7124 cells, marked with SKAP1, CD3E, and IKZF1) (Figure 1C). We calculated the inter‐site and inter‐patient proportions in each cluster to characterize the organ‐specific TME of LUAD (Figure 1D). In general, the proportion of immune cells was lower in patients with liver metastases. The proportions of B, plasma, and T cells were higher in primary LUAD and adrenal gland metastases than those in liver and brain metastases. Next, we performed the Ro/e analysis to evaluate the tissue enrichment of each cell population (Figure 1E). The Ro/e analysis was performed to quantify the enrichment of cell clusters across different anatomical sites based on the ratio of observed to expected cell numbers in each cluster, where the expected cell numbers for each cluster in different anatomical sites were obtained using the chi‐squared test.^[^ ^30^ ^]^ Plasma and B cells were preferentially enriched in adrenal gland metastases, and mast cells were relatively enriched in primary LUAD. Cycling and endothelial cells were preferentially enriched in brain and liver metastases, respectively.

The inter‐site heterogeneity of the immune microenvironment stimulated a deeper investigation of major immune cell types. Therefore, we re‐clustered the macrophages, B and plasma cells, and T cells separately. The expressions of canonical markers for each sub‐cluster are presented as dot plots (Figure 1F–H). We identified four macrophage subclusters defined as AFF3^+^, C1QB^+^, MRC1^+^, and SPP1^+^ macrophages, which have also been identified in previous studies.^[^ ^31^, ^32^, ^33^ ^]^ (Figure 1F). B and plasma cells clustered into B cells, IgA‐plasma, and IgG‐plasma cells (Figure 1G). T cells were categorized into activated, CD28^+^, cytotoxic, exhausted, ICOS^+^, and memory subsets (Figure 1H).

### 2.2 Organ‐Specific Profiling of Macrophages, B and Plasma Cells, and T Cells

**Figure 2:**
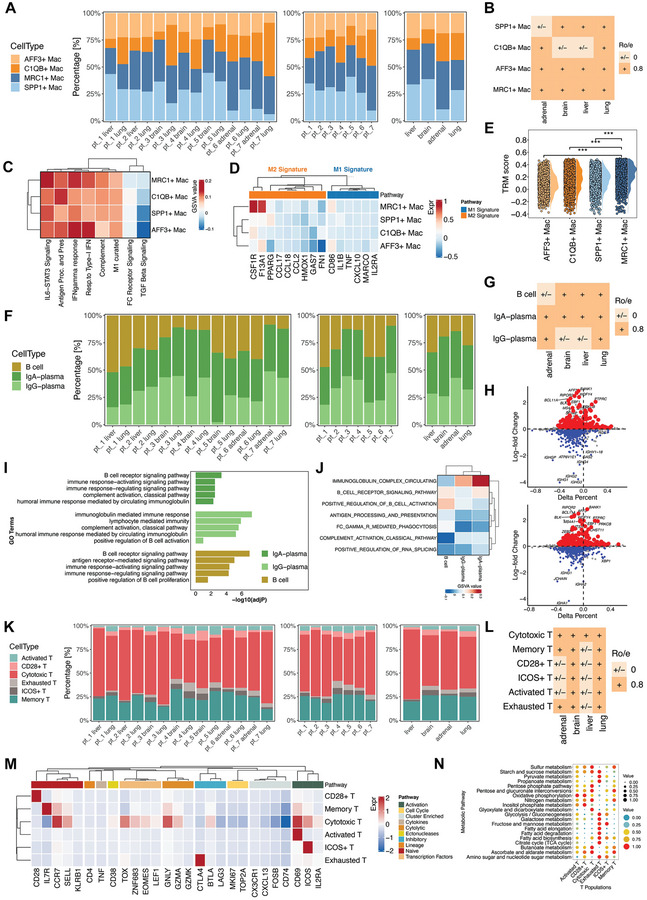
Diversity of macrophages, B and plasma cells, and T cells in the LUAD TME of different anatomical sites. A) The frequencies of the macrophage subclusters in each sample. B) Tissue prevalence of each macrophage subcluster estimated by the Ro/e analysis. C) The potential biological functions and relevant signaling pathways of the four macrophage subclusters were evaluated by the ssGSEA according to the hallmark gene sets. D) The enrichment of M1‐ and M2‐macrophage‐related pathways in the four macrophage subclusters. E) Half violin plots showing the tissue‐resident macrophage scores of the subclusters. F) The frequencies of the B and plasma cell subclusters in each sample. G) Tissue prevalence of each B and plasma cell subcluster estimated by the Ro/e analysis. H) The volcano plots show differently expressed genes of the IgA‐ and IgG‐plasma cells compared to the B cells. I,J) The potential biological functions and relevant signaling pathways of the B and plasma cell subclusters were evaluated by the GO and ssGSEA analyses according to the hallmark gene sets. K) The frequencies of the T cell subclusters in each sample. L) Tissue prevalence of each T cell subcluster estimated by the Ro/e analysis. M) The expression of canonical markers and relevant signaling pathways of the six T cell subclusters were evaluated by the ssGSEA based on hallmark gene sets. N) Dot plots of median metabolic pathway scores of the six T cell subclusters.

As macrophages, B cells, plasma cells, and T cells constituted the major proportion of the immune cells, we next explored the organ‐specific immune milieu of LUAD, mainly focusing on re‐clustered subsets of these cell populations. The distribution of the four macrophage subsets varied across sites and patients, and adrenal gland metastasis was associated with a lower frequency of SPP1^+^ macrophages (**Figure** **2**A,B). SPP1^+^ macrophages are immunosuppressive cells that predict poor response to PD‐1 blockades and dismal clinical outcomes.^[^ ^33^ ^]^ We further investigated the expression patterns and signaling pathways of these macrophage subsets using the single sample gene set enrichment analysis (ssGSEA) (Figure 2C). C1QB^+^ macrophages showed heightened antigen processing and presentation activities, and AFF3^+^ macrophages showed enriched pathways related to the type I interferon response. MRC1^+^ macrophages showed enrichment of IL6‐STAT3 signaling and IFN‐γ response pathways, consistent with previous reports.^[^ ^34^, ^35^ ^]^ Referring to the signature gene sets of the M1 and M2 polarization states, we found that MRC1^+^ macrophages exhibited a higher enrichment of M2 signatures, with particularly high expression of CSF1R and F13A1 (Figure 2D). As is known, CSF1R is closely associated with the differentiation and maintenance of tissue‐resident macrophages (TRM).^[^ ^36^, ^37^ ^]^ We then calculated TRM scores for each subset based on the core gene signatures, including TIDM4, LYVE1, FOLR2, CCR2, and MHC‐II, as previously described.^[^ ^38^ ^]^ As shown in Figure 2E, the MRC1^+^ macrophages had the highest TRM scores. Because the enrichment of MRC1^+^ macrophages was comparable across sites, we can infer that the frequency of TRMs in the TME was relatively constant across different anatomical sites of LUAD, although specific subtypes might vary. The organ‐specific distributions of B and plasma cells were also calculated, and IgG‐plasma cells were less likely to be enriched in brain and liver metastases (Figure 2F,G). Compared with B cells, IgA‐ and IgG‐plasma cells highly expressed IGH‐related gene sets (Figure 2H). Subsequent ssGSEA and GO analyses were performed to explore the potentially enriched signaling pathways and biological functions of the different subsets. IgA and IgG plasma cells were enriched in the pathways related to the circulating immunoglobulin complex. B cells showed the enrichment in B cell receptor‐related signaling pathways and heightened positive regulation of B cell activation (Figure 2I,J). These findings are consistent with the recognized functions of B and plasma cells, which present tumor antigens and produce tumor antigen‐specific antibodies respectively.^[^ ^39^ ^]^

We also delineated the inter‐site and inter‐patient distributions of T cell subsets, and Ro/e analysis was performed to resolve the organ‐specific enrichment of each subset (Figure 2K,L). We then investigated the variations in the expression patterns and signaling pathways among these T‐cell subsets (Figure 2M). Memory T cells highly expressed IL7R and CCR7. Cytotoxic T cells expressed high levels of GNLY, a cytotoxicity marker. The activated and ICOS^+^ subsets highly expressed CD69 and ICOS, respectively, and were enriched in the T cell activation pathway. CTLA4 was specifically expressed in the exhausted subset. ssGSEA was performed to explore the metabolic characteristics of these T‐cell subsets (Figure 2N). Oxidative phosphorylation (OXPHOS) was the top‐scoring pathway in the activated subset, consistent with previous findings that decreased OXPHOS expression can dampen the anti‐tumor functions of CD8^+^ T cells.^[^ ^40^, ^41^ ^]^ CD28^+^ and cytotoxic subsets were validated to have enriched pathways for inositol and pentose phosphate metabolism respectively.^[^ ^42^, ^43^, ^44^ ^]^ Consistent with previous studies, we found the exhausted subset was enriched in pathways related to glycolysis and fatty acid metabolism.^[^ ^44^, ^45^, ^46^ ^]^ The ICOS^+^ subset was associated with glycolysis and fatty acid biosynthesis.^[^ ^47^ ^]^ The memory subset had increased ascorbate metabolic activity.^[^ ^48^ ^]^ Collectively, these findings further confirmed the distinct architectures of the immune microenvironment and the diverse phenotypic states of immune cells across different sites of LUAD at high resolution.

### 2.3 Single‐Cell Analysis for Organ‐Specific Spatial Infiltrations in the TME

**Figure 3:**
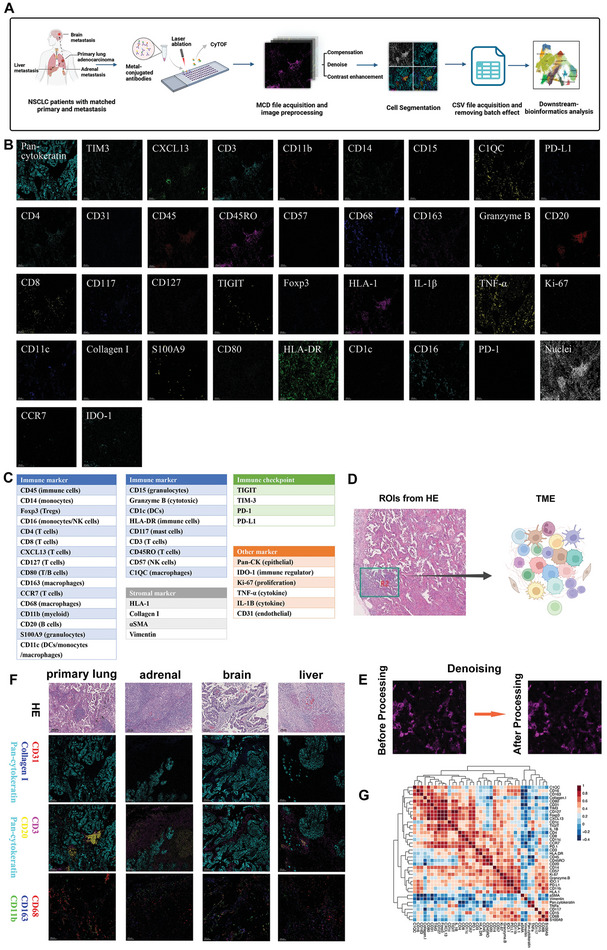
The imaging mass cytometry (IMC) technique reveals the spatial topology of metastatic LUAD TME at a single‐cell level. A) Schematic of the IMC pipeline applied to FFPE tissue samples from paired primary and different metastatic lesions. B) Single color staining of each marker. The antibody panels are listed in Table S3 (Supporting Information). C) Panel of all the markers used in the IMC. D) Regions of interest for IMC to dissect organ‐specific TME. E) The example of one paired raw IMC image and preprocessed image. F) Representative IMC images and corresponding H&E staining images in each anatomical site. Scale bars, 100um. Pan‐CK (cyan), collagen I (blue), and CD31 (red) were used to depict the structure of LUAD. Pan‐CK (cyan), CD20 (yellow), and CD3 (magenta) revealed the distribution of B and T cells in the LUAD. CD11b (green), CD163 (blue) and CD68 (red) characterized the resident and inflammatory macrophages. G) The correlation heatmap of the staining markers in the IMC.

The cell composition of the organ‐specific LUAD TME was depicted by scRandom‐seq analysis. To further decipher the spatial topology of the organ‐specific TME, we used a 40‐marker IMC to explore the spatial distribution of immune cells in the same patients (**Figure** **3**A). We stained the specimens with a 40‐plex antibody panel targeting various types of cells and immune checkpoints and then acquired high‐dimensional histopathological images for each specimen exhibiting typical structural markers such as pan‐cytokeratin (Pan‐CK) for epithelial cells, collagen I, and immune cell markers like CD20 and CD3 for B and T cells, respectively (Figure 3B,C). High‐resolution images were processed to resolve the TME (Figure 3D). To improve image quality, we preprocessed the scanned images after IMC before single‐cell segmentation, which consisted of compensation, denoising, and contrast enhancement according to published methods.^[^ ^49^, ^50^, ^51^ ^]^ As shown in Figure 3E, the image quality was significantly improved after preprocessing. We then examined the distinct organ‐specific spatial topology of LUAD. Pan‐CK^+^ epithelial cells, CD31^+^ endothelial cells, and deposited collagen I were clearly present in the scanned images, which was consistent with their spatial distribution in the corresponding hematoxylin and eosin (H&E) staining images (Figure 3F). Immune cells, such as T cells (CD3^+^) and B cells (CD20^+^), were mainly distributed in the tumor stromal area. Inflammatory (CD68^+^ CD11b^+^) and resident (C1QC^+^ CD163^+^ CD68^+^ CD11b^low^) macrophages were also clearly characterized using IMC (Figure 3F). The cells and components were further segmented using a connectivity‐aware segmentation method.^[^ ^52^, ^53^ ^]^ The expression of each marker was quantified and converted to an expression matrix. A correlation heatmap of the staining markers is presented in Figure 3G. Strong correlations were detected between CD20 and CD45 or CD45RO.

**Figure 4:**
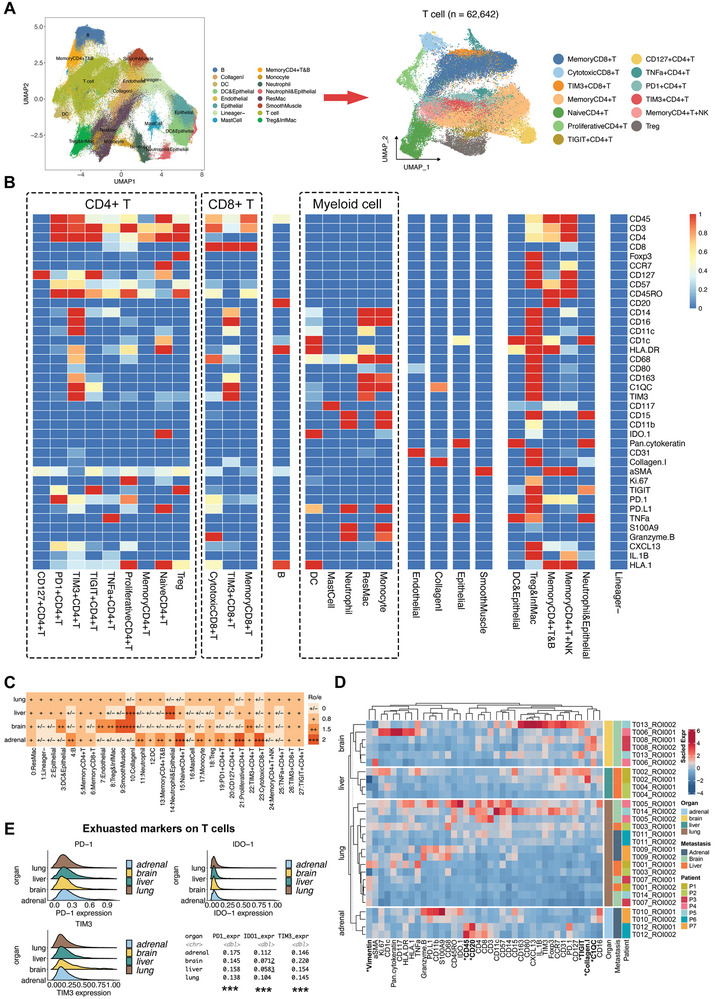
The divergent organ‐specific TME of LUAD detected by IMC techniques. A) The UMAP plots for major clusters and T cell subsets based on single‐cell data of 250 600 cells from IMC images. B) The heatmap presents the normalized expression levels of indicated markers for each cluster. C) Tissue prevalence of each cluster on IMC images estimated by the Ro/e analysis. D) The heatmap of scaled marker expressions in each specimen from different metastases. E) The expression levels of exhausted markers on T cells in each anatomic site of LUAD.

IMC images were segmented into 250 600 cells, which were clustered into 23 distinct immune cell clusters, along with endothelial cells, epithelial cells, smooth muscle cells, lineage‐negative cells, and collagen I (**Figure** **4**A; Figure S1A,B, Supporting Information). Theses cell clusters were annotated according to the expression of canonical markers, and the average expression of lineage markers for each cluster is shown in Figure 4B (Table S1, Supporting Information). The lineage‐negative cluster contained cells that could not be defined as a particular cell type. A single meta‐cluster could consist of multiple types of cells due to the close interaction of neighbor cells and the limited resolution of IMC such as regulatory T cells (Tregs) and inflammatory macrophages (InfMac). The limited resolution of IMC occurs mainly in regions with high cell densities, leading to the detection of overlapping markers. This artifact persisted throughout subsequent analyses and could not be overcome by increasing the resolution of IMC. A similar marker overlap also exists in multiplex immunohistochemistry imaging despite having a higher resolution limit. However, such an overlap likely signified the close interaction of neighboring cells and did not impede the conclusions drawn in this study. The proportion of each cluster relative to the total number of cells was calculated and plotted (Figure S1C, Supporting Information). Resident macrophages (ResMac), memory CD4^+^ T, memory CD8^+^ T, and B cells were the top four immune cell populations. The clusters were assigned to each specimen. The fractions of each cluster in all the specimens are presented as histograms (Figure S1D, Supporting Information). These clusters were distributed divergently across different anatomical sites and patients (Figure S2A–D, Supporting Information). Moreover, the differential patterns of these clusters between primary and metastatic lesions varied across individuals, even in those with the same type of metastatic lesion (Figure S2E, Supporting Information). In addition, moderately differentiated LUAD had higher proportions of ResMacs, clusters of Tregs and InfMacs, B cells, memory CD4^+^ T cells, and clusters of memory CD4^+^ T and B cells than poorly differentiated LUAD (Figure S2F, Supporting Information).

Ro/e analysis was performed to quantify the organ‐specific enrichment of these clusters (Figure 4C). Cytotoxic CD8^+^ T cells, proliferative CD4^+^ T cells, naïve CD4^+^ T cells, B cells, neutrophils, clusters of memory CD4^+^ T and B cells, monocytes, and PD‐1^+^ CD4^+^ T cells were preferentially enriched in adrenal gland metastases. Clusters of dendritic cells (DCs) and epithelial cells, endothelial cells, smooth muscle cells, TIM3^+^ CD4^+^ T cells, and clusters of Tregs and InfMacs were enriched in brain metastases. Liver metastasis showed enrichment of the cluster consisting of neutrophils and epithelial cells. Moreover, collagen I tended to be deposited in the brain and liver metastases. We also evaluated and compared the expression levels of these markers across different metastatic anatomical sites. The expression levels of vimentin, CD45, CD20, TIGIT, collagen I, and C1QC were significantly different across the four distinct anatomical sites (Figure 4D). The organ‐specific expression levels of exhausted T‐cell markers are shown in Figure 4E. T cells in the adrenal gland metastasis showed significantly higher PD‐1 expression. In contrast, the brain and liver metastases expressed higher levels of TIM‐3. We further compared the frequencies of each cluster among different anatomical sites (Figure S3, Supporting Information). In general, the distribution patterns of cell clusters and stromal matrices could be divided into two categories. Specifically, a similar tendency was observed in primary lung and adrenal gland metastatic lesions, whereas brain and liver metastases had similar immune milieu. The primary lung and adrenal gland metastatic lesions had significant enrichment of B cells and the cluster of memory CD4^+^ T and B cells, and the primary LUAD also had a higher frequency of TNF‐α^+^ CD4^+^ T cells. In summary, we spatially resolved the organ‐specific architecture of the TME in LUAD using the 40‐marker IMC and further validated the findings of the snRandom‐seq analysis.

### 2.4 Cellular Neighborhoods Revealing the Organ‐Specific Immune Spatial Topology

**Figure 5:**
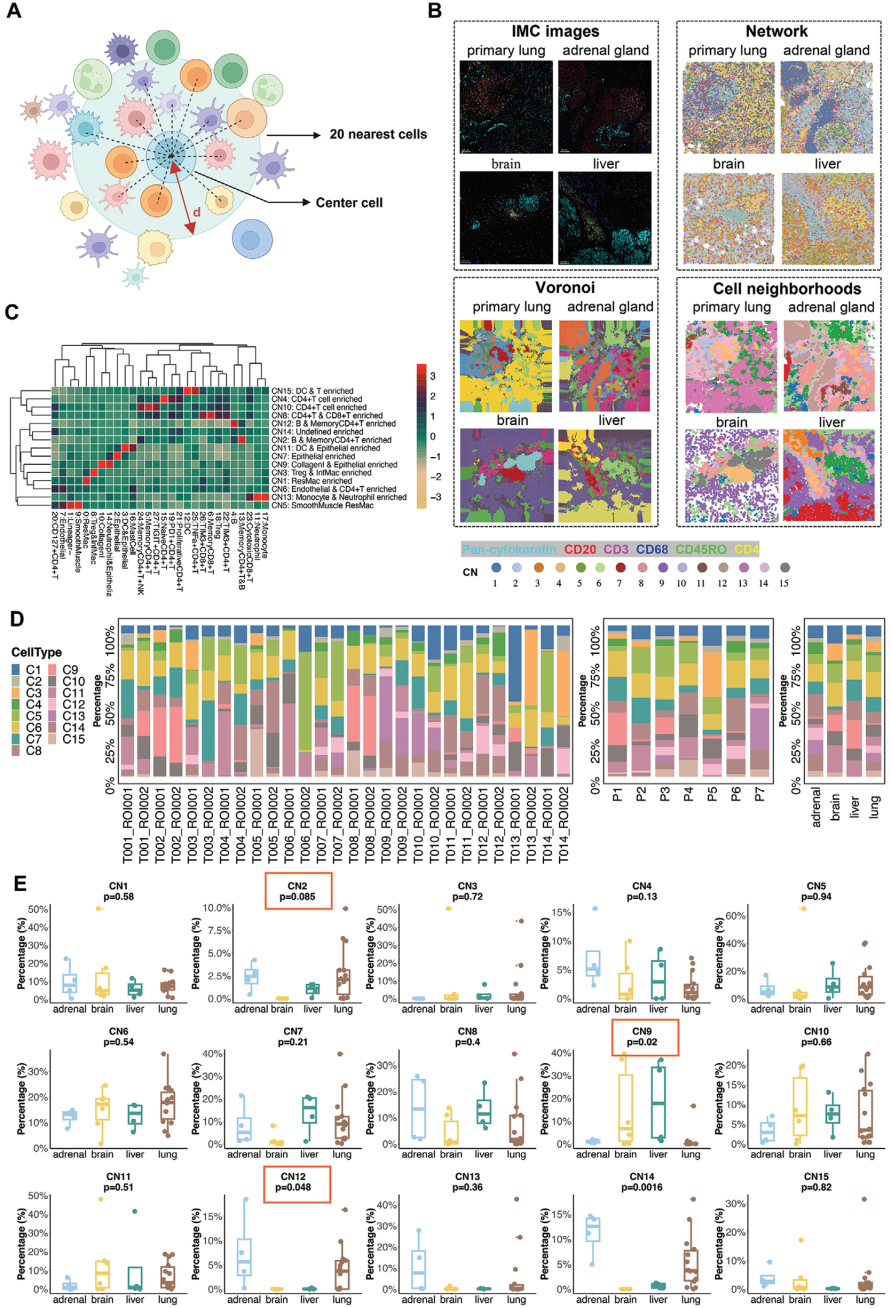
Tissue neighborhood analysis reveals organ‐specific compositions of cellular neighborhoods (CNs). A) Schematic demonstrating the identification of CNs. The CN was defined based on the center cell and its nearest 20 cells. B) Representative Network, Voronoi, and CN diagrams of TME in the four anatomical sites of LUAD with the corresponding IMC images. C) Identification of 15 distinct CNs from the 27 cell clusters along with collagen I and their corresponding abundances in each CN. D) The frequency of each CN presented as a proportion in each sample. E) The distribution of each CN across different anatomical sites of LUAD based on IMC results. The Wilcoxon rank‐sum tests were adopted to evaluate the statistical significance.

Studies have highlighted that complex interactions among different cells deeply influence tumor biology.^[^ ^54^, ^55^ ^]^ Thus, we performed a regional cellular neighborhood analysis to reveal multicellular structures within the LUAD at different anatomical sites. We defined the cell neighborhood (CN) as the nearest 20 cells to the center cell (**Figure** **5**A). To visualize organ‐specific cell interactions and functional units in the TME of LUAD, network, Voronoi, and CN patch plots were constructed for each IMC image (Figure 5B). A total of 15 CNs were identified and annotated based on the major cell types (Figure 5C). The cellular compositions of these CNs fully recapitulated the architecture of the TME, including epithelial‐cell enriched spots with co‐existence of DCs (CN11) or collagen I (CN9), two immune hotspots with enrichment of B cells and memory CD4^+^ T cells (CN2 and CN12), ResMacs‐enriched (CN1), Tregs and InfMacs‐enriched (CN3), CD4^+^ T cell‐enriched with (CN8) or without (CN4 and CN10) co‐existence of CD8^+^ T cells, and a CN mainly composed of lineage‐negative cells (CN14). The spatial topology of IMC was represented by CN patch plots, with various CNs indicated by different colors (Figure S4, Supporting Information). As shown in Figure 5B, the spatial cell interactions and CN functional units presented in the network, Voronoi, and CN patch diagrams matched well with the IMC images at each LUAD anatomical site.

The distribution of each CN across different anatomical sites of LUAD and patients is presented in histograms (Figure 5D). Next, we compared the enrichment of each CN across different anatomical sites of LUAD (Figure 5E). The frequencies of CN2 and CN12, both of which were enriched with B and memory CD4^+^ T cells, were significantly higher in primary lung and adrenal gland metastatic lesions than in brain and liver metastases. Moreover, brain and liver metastases showed significant enrichment in CN9 (collagen I and epithelial cells enriched). The distribution patterns of CNs in the primary and metastatic lesions within one patient varied according to the type of metastasis (Figure S5A,B, Supporting Information). When classified by the type of metastases, we observed that CN2 and CN12 (B and memory CD4^+^ T cells enriched), CN4(CD4^+^ T cells enriched), CN8 (CD4^+^ and CD8^+^ T cells enriched) and CN13 (monocytes and neutrophils enriched) were mainly from the adrenal gland metastases (Figure S5C, Supporting Information). The CNs with enrichment of PD‐1^+^ T cells (CN4 and CN8) are mainly enriched in the adrenal gland metastasis. Furthermore, the landscape of CNs varied among the LUAD patients with metastases to different organs (Figure S5D, Supporting Information). The organ‐specific spatial topologies of LUAD dissected by CN analysis were consistent with the diversity revealed by snRandom‐seq and IMC images. Adrenal gland metastases had the highest enrichment of B and CD4^+^ T cells, whereas these immune cells and their corresponding functional units were relatively absent in brain and liver metastases.

### 2.5 Organ‐Specific Enrichment of Tertiary Lymphoid Structures

**Figure 6:**
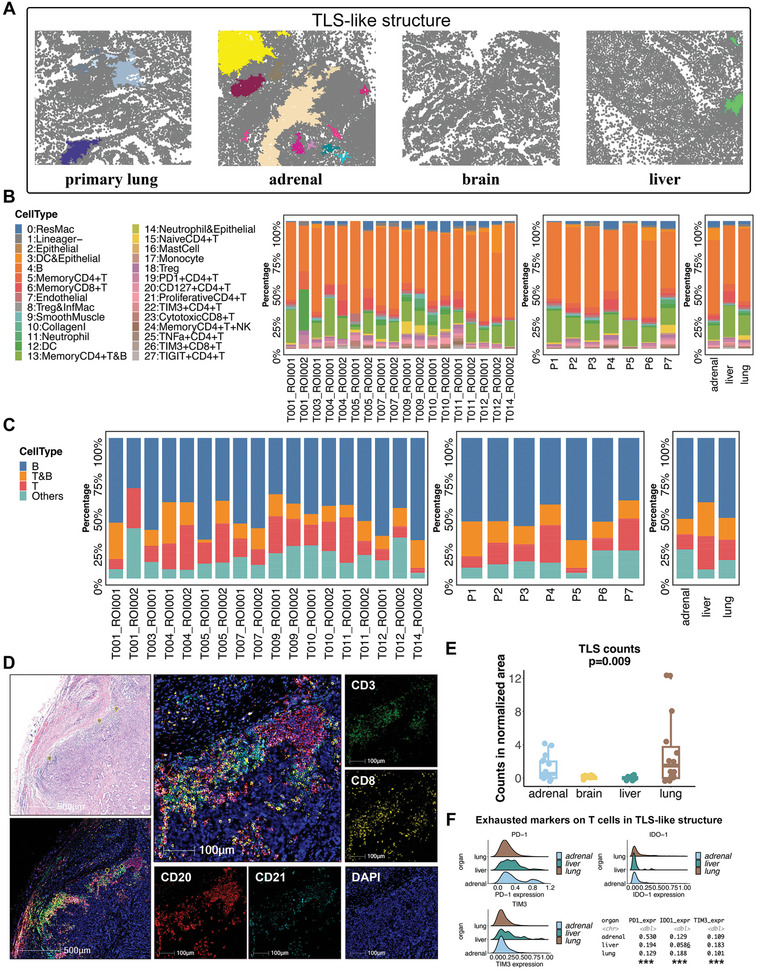
Divergent densities of the tertiary lymphoid structure (TLS) in different anatomical sites of LUAD. A) Representative patch diagrams of the TLS‐like structure in the four anatomical sites of LUAD. Each color represents one TLS‐like structure. B) The frequencies of each cell cluster identified from IMC images in the TLS‐like structures in each sample. C) The frequencies of T‐cell and B‐cell‐related clusters identified from IMC images in the TLS‐like structures in each sample. D) The presence of TLS identified by the H&E staining and multiplex immunofluorescence (mIF) staining of CD3, CD8, CD20, and CD21. E) Densities of TLS in different anatomical sites of LUAD from the validation cohort. The P value was estimated by the Wilcoxon rank‐sum test. F) The expression levels of exhausted markers on T cells in TLS‐like structures in each anatomic site of LUAD.

The concomitant enrichment of B and CD4^+^ T cells stimulated our attention to the tertiary lymphoid structure (TLS). We then performed patch analysis to identify TLS‐like structures in the IMC images (**Figure** **6**A; Figure S6, Supporting Information). The densities of TLS‐like structures in the TME varied across different anatomical sites in LUAD. Adrenal gland metastases had the most abundant TLS‐like structures, followed by primary lung tumors, whereas no TLS‐like structures were detected in brain metastases. To further dissect the composition of the TLS‐like structures, we calculated the proportion of cell clusters in these TLS‐like structures. B cells were the most common cell type in the TLS‐like structures, which was conserved across the TLS‐like structures at different sites. Memory CD4^+^ and CD8^+^ T cells, and the cluster of memory CD4^+^ T and B cells also accounted for substantial proportions (Figure 6B,C). The distribution of cell clusters was similar in the TLS‐like structures across different sites, indicating the preserved essence of the TLS‐like structure in primary and metastatic LUAD (Figure S7, Supporting Information). To validate these results, we constructed another cohort of 43 patients with metastatic LUAD. To detect TLS at different anatomical sites of LUAD based on surgical samples, H&E, immunohistochemistry (IHC) (CD3, CD8, CD20, and CD21), and multiplex immunofluorescence (mIF) staining (including CD3, CD8, CD20, CD21, and DAPI) were simultaneously performed for each specimen (Figure 6D; Figure S8, Supporting Information). We validated that the TLS was significantly more enriched in primary LUAD and adrenal gland metastases than in brain and liver metastases (Figure 6E). We further evaluated the expression levels of exhausted T‐cell markers in TLS‐like structures and found that the level of TIM‐3 was markedly higher in liver metastases (Figure 6F). Taken together, snRandom‐seq revealed divergent enrichment of B, plasma, and T cells across different sites of LUAD, and the spatial proximity of these cell clusters was detected by IMC, which was further validated by varying densities of TLS in different anatomical sites based on H&E, IHC, and mIF staining.

### 2.6 Cell Interactions at Spatial and Molecular Levels

**Figure 7:**
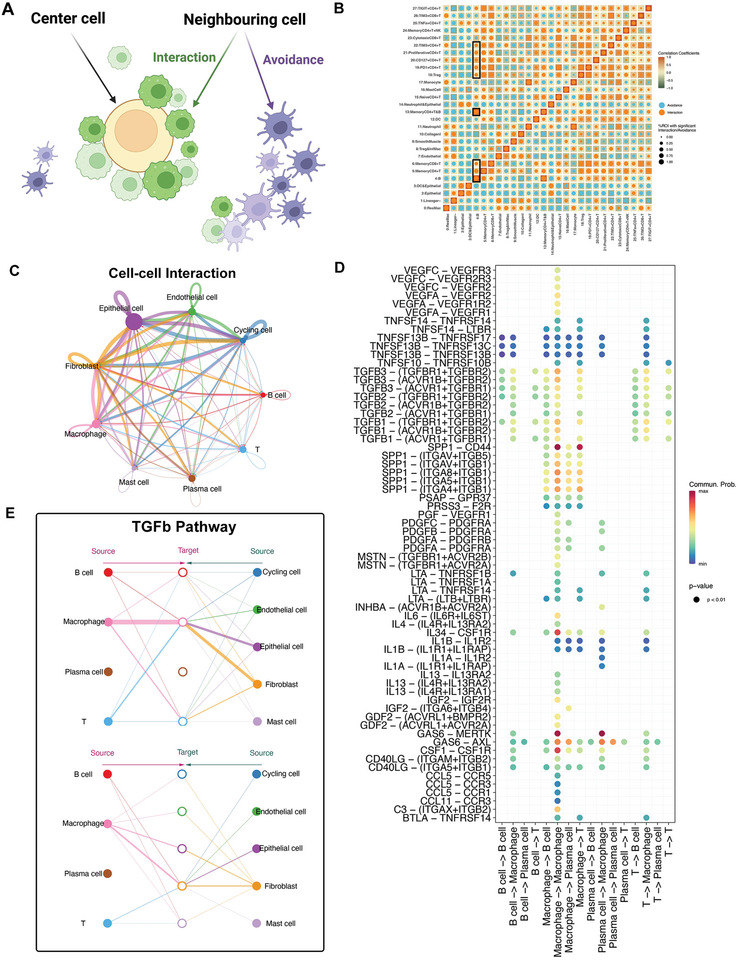
Spatial and molecular cellular communication network in the TME of LUAD. A) Schematic of spatial cell‐cell interaction analysis. The interaction patterns could be classified into the interaction pattern and avoidance pattern. B) Neighborhood analysis revealing the spatial cell interactions on IMC images. Rows represent the centered cell type and columns represent other cell types surrounding the center cell type. C) Different numbers and strengths of communications of the major cell types identified by the snRandom‐seq analysis. D) Dot plots showing the intricate interactions in terms of specific ligand‐receptor pairs among the B cells, macrophages, plasma cells, and T cells. E) Hierarchical plots showing the inferred cellular communication network for TGF‐β signaling.

To explore the mechanisms underlying the organ‐specific TME, we performed a regional correlation analysis to distinguish spatial interaction and avoidance pairs in the LUAD topology (**Figure** **7**A). The B cells interacted frequently with the memory CD4^+^ and CD8^+^ T cells, the cluster of memory CD4^+^ T and B cells, TIM‐3^+^, proliferative, and CD127^+^ CD4^+^ T cells. Avoidance patterns were observed between these cell types and the epithelial cells (Figure 7B). These spatial interaction modes to some extent also confirmed the existence of TLS, an ectopic lymphoid organ composed of B cells, T cells, and myeloid DCs.^[^ ^56^ ^]^ Next, we performed a cell‐cell interaction analysis to investigate the communications among major cell types (Figure 7C). We focused on the cellular communications among the major immune cell types, including B cells, macrophages, plasma cells, and T cells (Figure 7D). Interactions between macrophages and other cell types were mediated mainly by the SPP1 ligand. In addition, TGF‐β‐related signaling pathways were the dominant mediators of cellular communications among B cells, macrophages, and T cells; however, these signaling pathways were barely involved in the interactions of plasma cells.

TGF‐β is recognized as the dominant mediator of the immunosuppressive microenvironment.^[^ ^57^ ^]^ We then performed network centrality analysis to explore the intercellular communication network for TGF‐β signaling (Figure 7E). B cells, macrophages, and T cells regulate each other through TGF‐β signaling pathways, and macrophages could be the gatekeepers of these cell‐cell communications. The TGF‐β produced by macrophages can suppress T cell responses, and the TGF‐β antibody could restore T cell cytotoxicity.^[^ ^58^ ^]^ Activated B cells could also impair Th1 immunity or cause anergy of CD8^+^ T cells by expressing TGF‐β.^[^ ^59^, ^60^ ^]^ The fibroblast was also the major source of TGF‐β ligands acting on immune cells, especially on the macrophages. These findings are consistent with known sources of TGF‐β, which mainly include cancer‐associated fibroblasts, myeloid cells, and cancer cells.^[^ ^61^, ^62^, ^63^ ^]^ In summary, these results suggested the complex and diverse communication across B cells, macrophages, T cells, and other stromal cells, among which the TGF‐β‐related signaling pathway was a vital mediator.

## 3 Discussion

Despite the recognized associations between metastases at different sites and clinical outcomes, current treatment guidelines barely incorporate organ‐specific treatment paradigms, owing to the lack of a comprehensive understanding of site‐specific behaviors. In the present study, we originally deciphered the heterogeneous atlas of the TME in paired primary LUAD and metastases in different organs at single‐cell resolution while preserving the architecture to depict organ‐specific spatial topology. We identified various compositions of major immune cells and different molecular interaction patterns in the TME of LUAD at four different anatomical sites, including the lung, brain, liver, and adrenal gland. Furthermore, the IMC revealed divergent cellular spatial positioning and interactions at these distinct anatomical sites. Specifically, adrenal gland metastases exhibited enrichment of B and T cells, which were also spatially gathered and further formed TLSs, whereas brain and liver metastases had significantly more deposited collagen I. In addition, we detected higher expression of TIM‐3 in brain and liver metastases, albeit with comparable expression levels of PD‐1 across the four anatomical sites of LUAD. Overall, this study revealed the potential mechanism for the distinct response patterns to PD‐1 blockade in LUAD patients with metastases at different sites, which indicates the necessity for precise immunotherapy strategies or locoregional treatments for specific metastatic sites, such as the brain and liver.

While cancers arising from different organs exhibit varying susceptibilities to immunotherapy, the effectiveness of immunotherapy can starkly differ among metastatic patients with identical cancer types owing to anatomical differences in metastases. Notably, patients with brain or liver metastases have worse clinical outcomes owing to poorer responses to immunotherapy or chemotherapy than those with metastases to other anatomical sites, such as the lungs, lymph nodes, or adrenal glands.^[^ ^10^, ^13^, ^14^ ^]^ According to the “seed and soil” hypothesis, the intrinsic properties of tumor cells and specific niches of metastatic sites together form a unique organ‐specific metastatic TME.^[^ ^64^ ^]^ Brain and liver metastases are commonly associated with poor response to immunotherapy in various types of cancers, such as lung cancer and melanoma.^[^ ^13^, ^65^ ^]^ With the development of high‐throughput scRNA‐seq, the single‐cell profile and therapy‐induced evolution of primary lung tumors have been extensively characterized.^[^ ^66^, ^67^, ^68^ ^]^ Although the underlying mechanisms of resistance to PD‐1 blockade have been well elucidated in primary tumors, organ‐specific immunoregulation of metastases is underappreciated, especially at the single‐cell and spatial levels. The unique ecosystem of metastatic LUAD has barely been elucidated, which is essential for guiding organ‐specific treatment approaches in the context of divergent response patterns to ICIs among patients with metastases at different sites. Elucidating the tumor organ‐specific immune context has been recognized as one of the top ten challenges in cancer immunotherapy.^[^ ^24^ ^]^

Although divergent immune microenvironments have been detected between brain metastases and paired primary LUAD, current studies investigating the TME of metastatic LUAD are mostly conducted at low resolution using bulk RNA or whole‐exome sequencing because of the challenges in acquiring qualified samples from different metastatic sites for scRNA‐sequencing.^[^ ^69^, ^70^ ^]^ In addition, dissecting the intrinsic properties of organ‐specific TME requires treatment‐naïve tissues because systemic treatments can dramatically remodel the TME. Current clinical guidelines recommend systemic treatment as the first‐line therapy for patients with metastatic lung cancer, and biopsies for metastatic lesions are not routinely required when primary LUAD has been pathologically confirmed. In this context, it is exceedingly challenging to acquire treatment‐naïve paired primary LUAD and metastases in different organs, not to mention the requirement of fresh tissue samples in most scRNA‐seq methods. FFPE tissue specimens are currently optimal candidates for collecting these rare paired surgical samples. Nevertheless, the isolation of single intact cells or nuclei and the extraction of RNA from FFPE tissues remain challenging owing to issues such as RNA cross‐linking and degradation.

Recently, the development of snRandom‐seq has enabled researchers to profile single nuclei transcriptomes from FFPE tissues at single‐cell resolution.^[^ ^29^ ^]^ Using this pathology‐friendly technique, we performed high‐resolution analyses based on FFPE tissue samples from paired treatment‐naïve primary LUAD and metastases in the brain, liver, and adrenal gland. Multiple major cell types were identified, including B cells, cycling cells, endothelial cells, epithelial cells, fibroblasts, macrophages, mast cells, plasma cells, and T cells. The abundance of these cell types varied across different anatomical sites of LUAD. The adrenal gland metastases were infiltrated with more immune cells, especially B and plasma cells, whereas the brain and liver metastases were less immunogenic but deposited more collagen I. Compared with the considerable attention given to T cells in the immune biology of lung cancer, the profiling of tumor‐infiltrating B and plasma cells is relatively understudied. Studies have reported that intratumoral B and plasma cells are positively related to the response and survival of patients receiving PD‐1 blockade in various cancers, such as NSCLC and melanoma.^[^ ^71^, ^72^ ^]^ Since several studies have reported that adrenal gland metastases and primary lung tumors response better to PD‐1 blockade than brain and liver metastases, we can infer that different densities of B and plasma cells in these anatomical sites could substantially contribute to divergent organ‐specific responses to immunotherapy.^[^ ^10^, ^13^, ^14^ ^]^ According to the results of the Ro/e analyses, the disparity in plasma cell enrichment between the adrenal gland metastases and brain or liver metastases was more significant than the difference in B cell enrichment. Plasma cells have a stronger predictive association with immunotherapy response than B cells.^[^ ^67^, ^71^ ^]^ Therefore, the plasma cell signature may be a precise biomarker for predicting the efficiency of the PD‐(L)1 blockade. We also identified a tissue‐resident subset of macrophages, MRC1^+^ macrophages, which highly expressed CSF1R and F13A1 and were enriched in the TME of LUAD. The T cell‐dependent release of CSF‐1 can increase the secretion of granulin from macrophages and cause adaptive resistance to PD‐1 blockade, indicating the potential application of CSF‐1 blockade to restore anti‐tumor responses.^[^ ^73^, ^74^ ^]^ It is well recognized that the TGF‐β signaling pathway acts as a major suppressor of both innate and adaptive immune responses in the TME.^[^ ^57^ ^]^ Using cell‐chat analyses, we detected that the TGF‐β signaling pathway was the dominant mediator for the cellular interactions among B cells, macrophages, and T cells, and the fibroblast was the major source of TGF‐β ligands.

In addition to the molecular and cellular composition of the TME, spatial distributions, and neighborhood communications constitute distinct immune niches and are correlated with immunotherapy responses.^[^ ^75^ ^]^ However, previous studies have barely investigated the spatial topology of the TME in different anatomical sites of LUAD at a single‐cell resolution. In this study, we used a 40‐marker IMC to address this issue. Compared to brain and liver metastases, primary lung tumors and adrenal gland metastases exhibited significant enrichment in B cells and memory CD4^+^ T cells. Additionally, the latter were enriched in proliferative CD4^+^ T and cytotoxic CD8^+^ T cells. Neighborhood analysis successfully reclassified these cells into 15 distinct CNs, capable of recapitulating organ‐specific TME. The proximity between B cells and memory CD4^+^ T cells demarcated CN2 and CN12, which were significantly enriched in primary lung sites and adrenal gland metastases. The presence of B cell‐rich niches indicates the potential formation of TLS, where B cells can improve antigen presentation and release tumor‐specific antibodies.^[^ ^56^ ^]^ The patch analysis in this study also detected the most abundant TLS‐like structures in adrenal gland metastases, which was further validated in another LUAD cohort. TLSs are associated with a better response to immunotherapy and prolonged survival in various tumor types.^[^ ^76^ ^]^ However, previous studies have mainly evaluated the densities of TLSs at the primary tumor site, and it remains unclear whether the densities differ between primary and metastatic tumors. In the present study, we initially evaluated the organ‐specific densities of TLSs and found different enrichments of TLSs in the surgical tissue samples from paired primary LUAD and metastases in different organs, including the brain, liver, and adrenal gland. Consistent with previous reports, metastases responding better to the PD‐1 blockade had higher densities of TLSs.^[^ ^71^ ^]^

The immune checkpoint therapy, represented by the PD‐1 blockade, has demonstrated efficacy against metastatic lung cancer. However, patients with brain or liver metastases respond poorly to PD‐1 blockade therapy. In the present study, we detected that the brain and liver metastases expressed higher levels of TIM‐3, another common T cell inhibitory receptor. Studies have found that high TIM‐3 expression serves as a specific marker for highly dysfunctional or terminally exhausted T cells and indicates a poor prognosis in cancer patients.^[^ ^77^, ^78^ ^]^ Blocking TIM‐3 alone or in combination with PD‐(L)1 blockade can enhance the efficiency of anti‐tumor immunotherapy.^[^ ^77^, ^79^ ^]^ In addition, upregulation of TIM‐3 is associated with resistance to PD‐1 blockade in NSCLC patients.^[^ ^80^ ^]^ These preclinical findings were further validated in the clinical trial, where the combination of anti‐TIM‐3 and anti‐PD‐1 has demonstrated good efficacy in patients previously resistant to PD‐1 blockades.^[^ ^81^ ^]^ Thus, the low density of TLSs might not be the only reason for the poor response to immunotherapy in patients with brain or liver metastases, and the co‐expression of multi‐inhibitory receptors is another essential issue. Moreover, phenotypic differences in TLS characterized by different expression levels of immune checkpoints have divergent effects on the prognosis of cancer patients.^[^ ^82^ ^]^ Similarly, we also detected the upregulation of TIM‐3 in the T cells of TLSs in liver metastases, which could be another potential mechanism underlying the poor response to immunotherapy. Our results indicate that dual blockade of PD‐1 and TIM‐3 may be a suitable treatment modality for LUAD patients with brain or liver metastases. Further subgroup analyses of the ongoing clinical trials (NCT03708328 and NCT04931654) will inspect this hypothesis.

This study had several limitations. First, the selected regions of interest (ROIs) for each specimen were used for IMC, and whether these ROIs could represent the heterogeneous organ‐specific TME needed to be validated. Thus, we selected two ROIs for each FFPE specimen by the experienced pathologist, mainly focusing on the regions with the most abundant immune cell infiltration. Moreover, the sequencing data from snRandom‐seq captured the transcriptome data from intact specimens, which compensated for the issue of the representativeness of IMC. Second, owing to the limitation of snRandom‐seq, it was difficult to classify CD4^+^ and CD8^+^ T cells as both markers are highly expressed in the cell membrane rather than in the nucleus. To solve this issue, we used IMC to further characterize the spatial topology of the T cell subpopulations. Third, the sample size was limited owing to the formidable challenge of obtaining surgical specimens from treatment‐naïve paired primary LUAD and metastases. We dissected the single‐cell profile of the TME at four different anatomical sites of LUAD at both cellular and spatial levels using snRandom‐seq and IMC. Biopsied tissue samples are usually insufficient for maintaining the homogeneity of the tissue samples used in these two technical methods. Moreover, the recommended first‐line treatments for metastatic lung cancer patients are systemic therapies that can remodel the tumor's immune microenvironment. Thus, acquiring sufficient treatment‐naïve tissue samples for high‐resolution analyses by surgical resection is extremely challenging, not to mention paired primary LUAD and metastases at different anatomical sites. Owing to the limited availability of samples, we were unable to proceed with paired testing of primary and metastatic lesions in this study. Further collection of surgical specimens is necessary to investigate the inter‐lesion immune heterogeneity between paired primary and metastatic lesions. In addition, more samples are needed to investigate the impact of differentiation status on the spatial topology of LUAD. Further validation is also warranted in the cohorts receiving single or dual ICIs with sufficient baseline tissue samples from different anatomical sites.

In conclusion, this study revealed the heterogeneous organ‐specific TME context in LUAD patients through a pathologically friendly strategy based on FFPE samples, not only in terms of cell types and communication but also in terms of spatial distributions and neighborhood interactions. These findings highlight the necessity for considering the anatomical sites of LUAD metastases when developing clinical treatment approaches.

## 4 Experimental Section

### Patients and Samples

The paired primary lung tumors and brain, liver, or adrenal gland metastases were collected from seven LUAD patients receiving surgery at the First Affiliated Hospital, Zhejiang University School of Medicine (FAHZU). The clinical characteristics of these patients are presented in Table S2 (Supporting Information). All surgical specimens analyzed in this study were treatment‐naïve. The tissue samples for scRandom‐seq and IMC were acquired from the tumor area. To validate the organ‐specific enrichment of TLS, surgical specimens were collected from different anatomical sites in another LUAD cohort, including the lungs (*n =* 13), brain (*n =* 12), liver (*n =* 7), and adrenal glands (*n =* 11). This study was approved by the Ethics Committee of FAHZU and was implemented in accordance with the standards of the Declaration of Helsinki (ethical number: IIT20230718A). Informed consent was obtained from all the enrolled patients or their immediate family members.

### SnRandom‐Seq

SnRandom‐seq was performed as previously described.^[^ ^29^ ^]^ The VITAcruizer Single‐Cell Preparation System V1.0 (M20 Genomics) was used for droplet generation, single‐cell encapsulation, and nucleic acid capture. The VITApilote High‐Throughput Eukaryotic Single‐Cell Transcriptome (Paraffin) Kit (M20 Genomics) was used for pre‐sequencing sample processing, single‐cell RNA library construction, and purification. The constructed single‐cell RNA library containing P5 and P7 primers was subsequently sequenced on the NovaSeq 6000 sequencing platform (Illumina). The default parameters of the STAR software were adopted to align the FASTQ file with the human reference genome (GRCh38). For each sample, gene‐barcode matrices were generated by tallying the unique molecular identifiers (UMIs) and removing non‐cell‐associated barcodes. Finally, the barcoded cells and their corresponding gene expression counts were merged to form a comprehensive gene barcode matrix.

### SnRandom‐seq Data Analysis

Quality control and integration were performed using the “Seurat” R package (version 4.4.0).^[^ ^83^ ^]^ Several steps were taken to filter out poor‐quality data. First, the genes covered by fewer than three cells were removed. Next, cells expressing < 500 or > 5000 genes and those containing < 400 or > 25 000 UMIs were filtered to exclude barcodes associated with empty partitions or doublet cells. Additionally, scDblFinder was utilized with default parameters to remove those with doublets and multiplets.^[^ ^84^ ^]^ Cells with mitochondrial content >15% were also removed. To integrate and embed the single cells from different individuals into a shared low‐dimension space, the integrated analysis was utilized in the Seurat v4 function “‘IntegrateData”’ which allowed us to perform batch effect correction and normalization. After generating the integrated matrix, an unsupervised graph‐based clustering algorithm was employed to cluster single cells using their expression, which was implemented in Seurat. The default parameters of Seurat were used unless specified otherwise. The UMI count matrix was normalized using the “NormalizeData” function with default parameters. Subsequently, a natural‐log transformed normalized gene expression matrix was used to identify 2000 highly variable genes via the “FindVariableFeatures” function with the “vst” method. These 2000 variable genes were then employed to cluster all cell types. Subsequently, 20 principal component analyses (PCA) were applied to the dataset to reduce dimensionality, after regressing the number of UMIs (counts). Finally, the “‘FindClusters”’ function was used on 20 PCs with a resolution of 1.2 to perform the first‐round cluster, and each cluster was annotated by known markers. Nonlinear dimensional reduction was performed using the Uniform Manifold Approximation and Projection (UMAP) method. To characterize each cluster, we utilized the “‘FindAllMarkers”’ procedure in Seurat. This method identifies markers based on log fold changes (FC) of mean expression and employs the Wilcoxon Rank‐Sum test by default (with parameters set as min.pct = 0.25 and logfc.threshold = 0.25). Feature genes along with known lineage‐specific markers were used for the cluster annotation. The “clusterProfiler” package (version 4.10.0) was utilized to conduct Gene Ontology (GO) analysis.^[^ ^85^ ^]^ The “GSVA” package (version 1.50.0) in R software was utilized to calculate the ssGSEA score for each gene set.^[^ ^86^ ^]^ To investigate the cell‐cell interactions, the ligand and target gene pairs were explored using the “cellchat” package (version 1.6.1) in R software.^[^ ^87^ ^]^

### IMC and Downstream Analysis

The ROIs for IMC were selected on the H&E slides by the experienced pathologist according to the same criteria, specifically focusing on the regions with the most abundant immune cell infiltration. After obtaining paraffin specimens from the enrolled patients and performing serial sectioning, one of the sections underwent H&E staining. Subsequently, the pathologist identified two regions infiltrated with the most abundant immune cells based on the pathological morphological structure revealed by the H&E staining. When observed under the light microscope at 400× magnification, numerous small clusters of predominantly blue‐stained cells were clearly visible, ranging from 1000 to 7000 square micrometers, indicating primarily lymphocytic infiltration. These immune cells were observed both at the periphery of the tumor tissue and within the tumor. Multiplexed images of the ROIs were obtained using the Hyperion Imaging System (Fluidigm). The ROIs were captured as square areas with a 400 Hz laser intensity. The acquired raw data were then pre‐processed according to the following steps: spillover signal compensation, image denoising, image contrast enhancement, and cell segmentation. The methods described above were based on a previously published study.^[^ ^88^ ^]^ A connectivity‐aware segmentation method was adopted for the segmentation of individual cells or components in various channels of IMC images.^[^ ^88^ ^]^ For cell segmentation, we used the region props function in MATLAB to identify connected components within the image. In the case of other membrane channels, artifacts were eliminated if their distance from the nearest nucleus centroids exceeded 15 pixels. Marker expression was normalized to the 99th percentile for each channel. The “Harmony” package (version 0.1.0) was used to correct batch effects. The “Rphenograph” (version 0.99.1) with 100 nearest neighbors was used for cell clustering. The cluster means were presented by a heatmap and were utilized for annotation. The “imcRtools” package (version 1.0.2) was used for downstream analysis. The CN of each cell was determined by identifying the 20 nearest neighboring cells based on the Euclidean distance. These neighbors were then clustered using K‐means clustering (*k* = 15) based on the 27 cell clusters, along with collagen I. The K‐means algorithm can efficiently cluster data points into groups based on similarity to provide insights into the underlying patterns, demonstrating faster execution times than hierarchical and model‐based clustering methods.^[^ ^89^ ^]^ The CNs were validated by overlaying the Voronoi diagrams on the corresponding original IMC images. To explore spatial cell‐cell interactions, the permutation test method from the “imcRtools” package (version 1.0.2) was employed to assess the interactions/avoidances between different cell clusters within each CN.^[^ ^90^ ^]^ The permutation test could be used to compare pairwise interactions between and within cell phenotypes to a random distribution. The comparison to a matched randomized tissue for each individual image controls both the distinct connectivity and specific cell types in that tissue.^[^ ^91^ ^]^

### H&E Staining

Tumor tissue sections (4 µm) were deparaffinized with xylene and rehydrated with graded alcohol. The sections were then rinsed three times with PBS and stained with hematoxylin for 30 min at room temperature. After washing with PBS, the sections were immersed in ammonia water to transform the nuclei form red to blue‐purple. The tissue slides were washed with 75% alcohol for two minutes. The cytoplasm was stained with eosin for one hour at room temperature. The sections were mounted after displacing anhydrous alcohol with xylene. A light microscope (Leica) was used to examine the sections and Image‐Pro Plus software (version 6.0) was used for image analysis.

### IHC Staining

Primary and metastatic LUAD tissues were fixed with 4% paraformaldehyde and embedded in paraffin. Tissue blocks were cut at 4‐µm thickness into four consecutive sections for IHC staining. Tissue sections were subsequently deparaffinized, and heat‐induced antigen retrieval was performed using Leica Epitope Retrieval 2 solution at 100 °C for 20 min. The tissue slices were blocked for 10 min at room temperature using a Leica blocking solution. The sections were incubated with primary antibodies against CD3 (1:200, Abcam), CD8 (1:100, Invitrogen), CD20 (1:100, Origene), and CD21 (1:200, SinoBiological) at room temperature for 25 min. Subsequently, LUAD tissue samples were treated with a peroxidase‐conjugated (HRP) secondary antibody (#DS9800, Leica) for 10 min. The Bond Polymer Refine detection system was used for visualization. All procedures were performed using an automated immunohistochemistry instrument (BOND RX, Leica). Slides were dehydrated, cleared, and covered with coverslips. The sections were scanned at 20× magnification using a Pannoramic 250 FLASH tissue imaging system (3D HISTECH).

### mIF Staining

mIF was conducted by staining 4‐µm‐thick FFPE whole LUAD tissue sections using standard primary antibodies sequentially and paired with tyramide system amplification (TSA) 5‐colors kit (H‐D110051‐50T, Yuanxibio). For subsequent rounds of staining (rounds two to five), the slides were washed in TBST buffer and then transferred to a preheated EDTA solution (90 °C) for heat processing using a microwave at 20% maximum power for 15 min. After cooling to room temperature in the same solution, the slides were incubated with an anti‐CD3 antibody (1:200, Abcam) for 60 min, followed by treatment with an HRP secondary antibody (#DS9800, Leica) for 10 min. Subsequently, labeling was performed for a strictly observed 10 min using TSA 480 according to the manufacturer's instructions. Throughout the process, the slides were washed with Tris buffer. The same procedure was repeated for the subsequent antibodies and fluorescent dyes in the following order: anti‐CD8 (1:100, Invitrogen)/TSA 520, anti‐CD20 (1:100, Origene)/TSA 570, and anti‐CD21 (1:200, SinoBiological)/TSA 620. Each slide was washed with distilled water and manually cover‐slipped. Nuclei were stained with DAPI Solution (Thermo Fisher Scientific, 62248) at 2 mg mL^−1^ for 10 min, washed in distilled water, and coverslipped manually. The slides were air‐dried and scanned at 20× magnification using a Pannoramic 250 FLASH tissue imaging system (3D HISTECH).

### Statistical Analysis

Statistical analyses were conducted using the R software (version 4.04). The appropriate statistical tests were selected based on data distribution and variability characteristics. Statistical significance was evaluated using the Kruskal–Wallis test, one‐way analysis of variance (ANOVA), and Student's t‐test. One‐way ANOVA was used to compare continuous variables with a normal distribution across multiple groups, while the Kruskal–Wallis test was employed to compare continuous variables with a non‐normal distribution among multiple groups. Furthermore, paired‐sample t‐tests were used to assess differences in continuous variables between the two groups. Data are presented as mean ± standard deviation. Statistical significance was defined as *p <* 0.05, denoted as *p < 0.05, **p < 0.01, ***p < 0.001, and ****p < 0.001.

## Conflict of Interest

The authors declare no conflict of interest.

## Author Contributions

X.S., X.T., C.L., and W.T. contributed equally to this work. X.B., Y.S., and X.S. designed the study. X.B., X.S., C.L., W.T., S.H., L.H., Y.Z., X.D., L.W., L.L., Yi.S., P.Z., and W.F. analyzed and interpreted the data. X.T. collected clinical samples. X.T. and Xiaodong.T. performed H&E and pathological evaluation. J.C. performed IHC, mIF, and pathological evaluation. X.B., X.S., and Y.J. performed the visualization work. X.S. and X.B. wrote the manuscript. W.F. and P.Z. edited the manuscript. All the authors read and approved the final manuscript.

## Supporting information

Supporting Information

## Data Availability

The data that support the findings of this study are available from the corresponding author upon reasonable request.
