# Supplementary material for: A Pathologically Friendly Strategy for Determining the Organ‐specific Spatial Tumor Microenvironment Topology in Lung Adenocarcinoma Through the Integration of snRandom‐seq and Imaging Mass Cytometry
Source: Adv Sci (Weinh). 2024 Apr 29;11(26):2308892. doi: 10.1002/advs.202308892 (PMC11234426; doi:10.1002/advs.202308892)
Supplement: Supplementary file 1 — Supporting Information [file ADVS-11-2308892-s001.pdf]

ADVANCED SCIENCE
Open Access

Supporting Information

for *Adv. Sci.*, DOI 10.1002/advs.202308892

A Pathologically Friendly Strategy for Determining the Organ-specific Spatial Tumor Microenvironment Topology in Lung Adenocarcinoma Through the Integration of snRandom-seq and Imaging Mass Cytometry

Xuqi Sun, Xiao Teng, Chuan Liu, Weihong Tian, Jinlin Cheng, Shuqiang Hao, Yuzhi Jin, Libing Hong, Yongqiang Zheng, Xiaomeng Dai, Linying Wu, Lulu Liu, Xiaodong Teng, Yi Shi, Peng Zhao, Weijia Fang, Yu Shi and Xuanwen Bao**

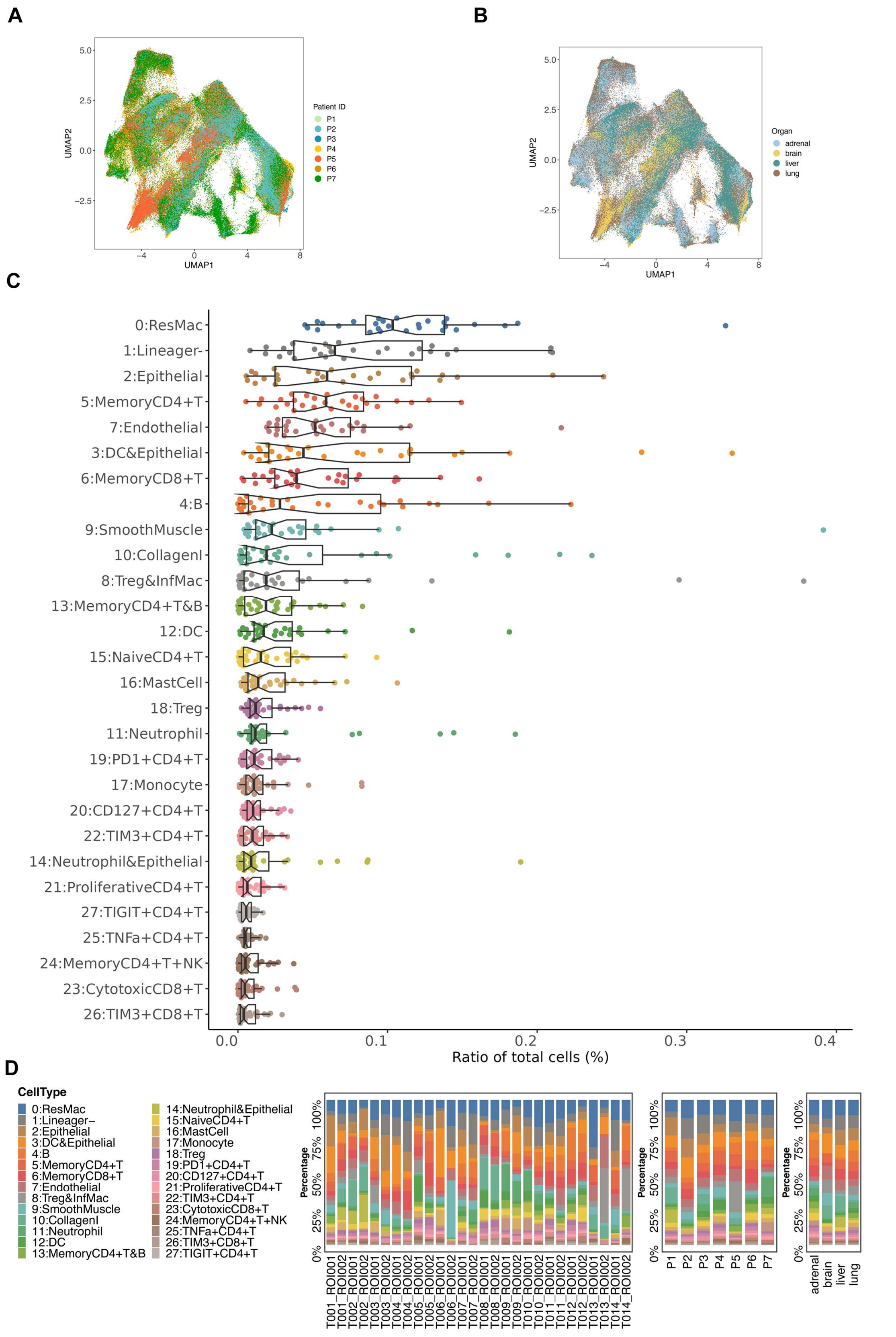

Figure S1. Organ-specific compositions of the cell clusters identified based on IMC images. **(A)** The UMAP plot based on IMC images colored by the patients. **(B)** The UMAP plot based on IMC images colored by the anatomical sites of LUAD. **(C)** The proportions of each cell cluster to the total cells on every IMC image. **(D)** The frequency of each cell cluster presented as a proportion of total cells in each sample.

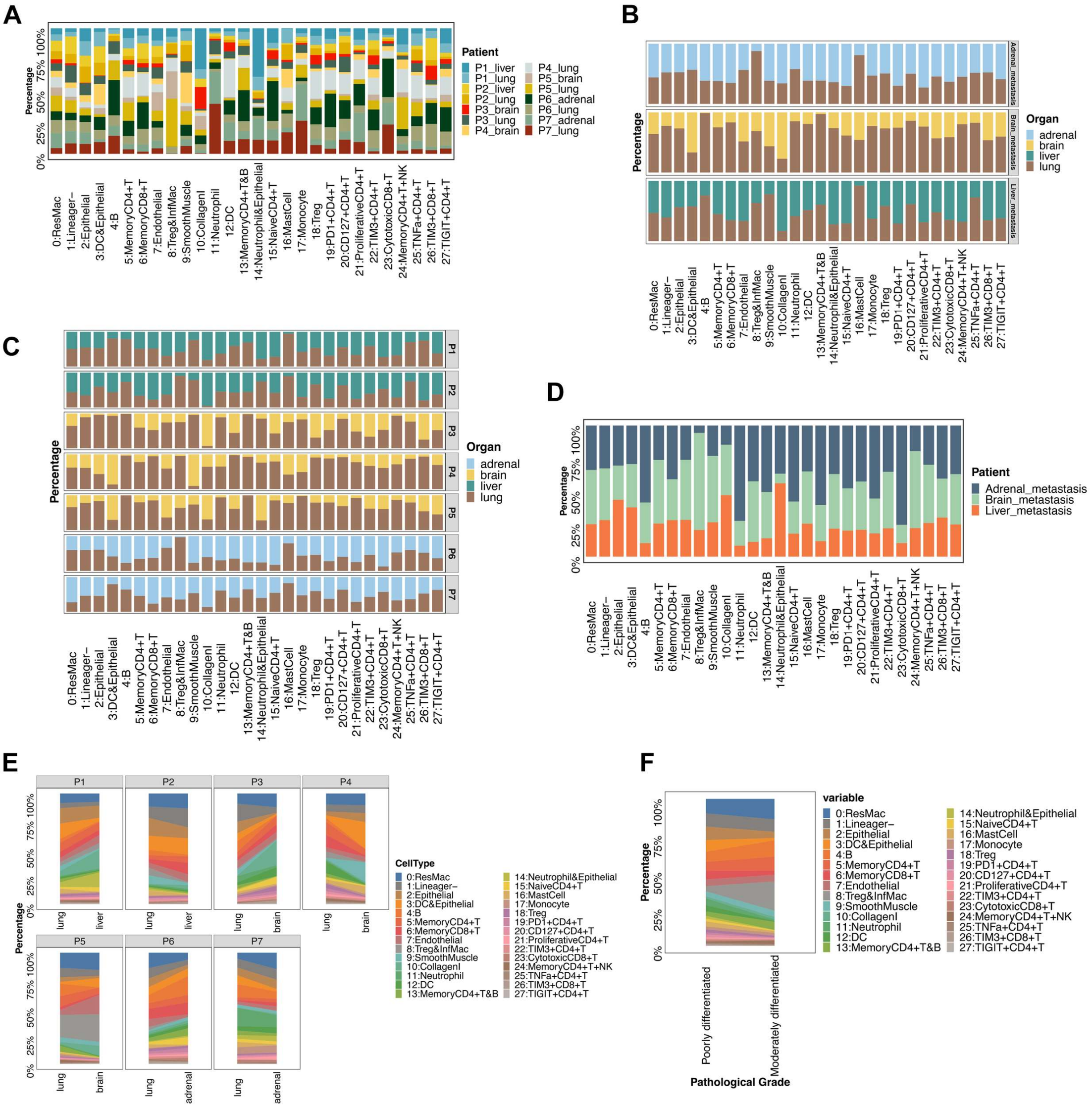

Figure S2. The frequency distribution patterns of the cell clusters on IMC images. **(A)** The distribution of each cell cluster in every anatomical site of the enrolled LUAD patients. **(B)** The distribution of each cell cluster in paired primary and metastatic LUAD classified by the anatomical site of metastases. **(C)** The distribution of each cell cluster in the paired primary and metastatic lesions of each patient. **(D)** The distribution of each cell cluster in the brain, liver and adrenal gland metastases. **(E)** The different distribution of cell clusters in the TME between primary LUAD and paired metastatic lesion in each patient. **(F)** The divergent TME components of moderately and poorly differentiated LUAD.

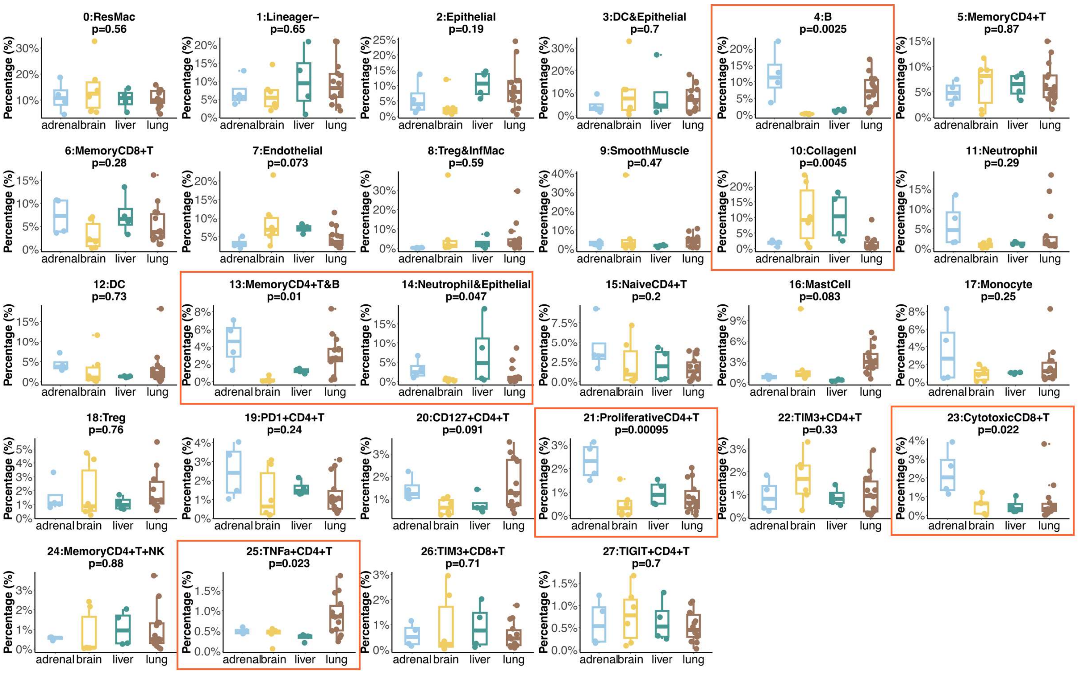

Figure S3. The distribution of each cluster across different anatomical sites of LUAD based on IMC results. The Wilcoxon rank-sum tests were adopted to evaluate the statistical significance.

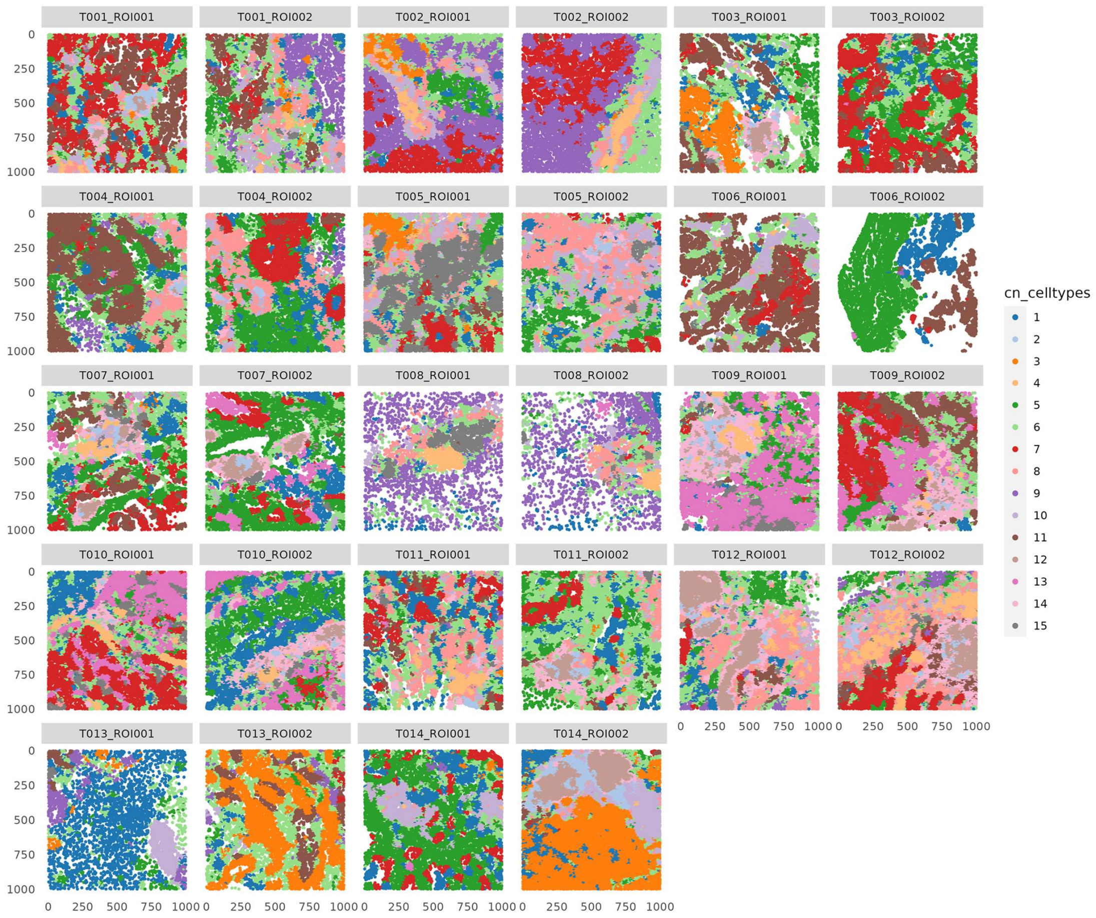

Figure S4. The cellular neighborhood patch diagrams of cellular neighborhoods in each sample based on the IMC. The corresponding tissue types of each sample in IMC are listed in Table S4.

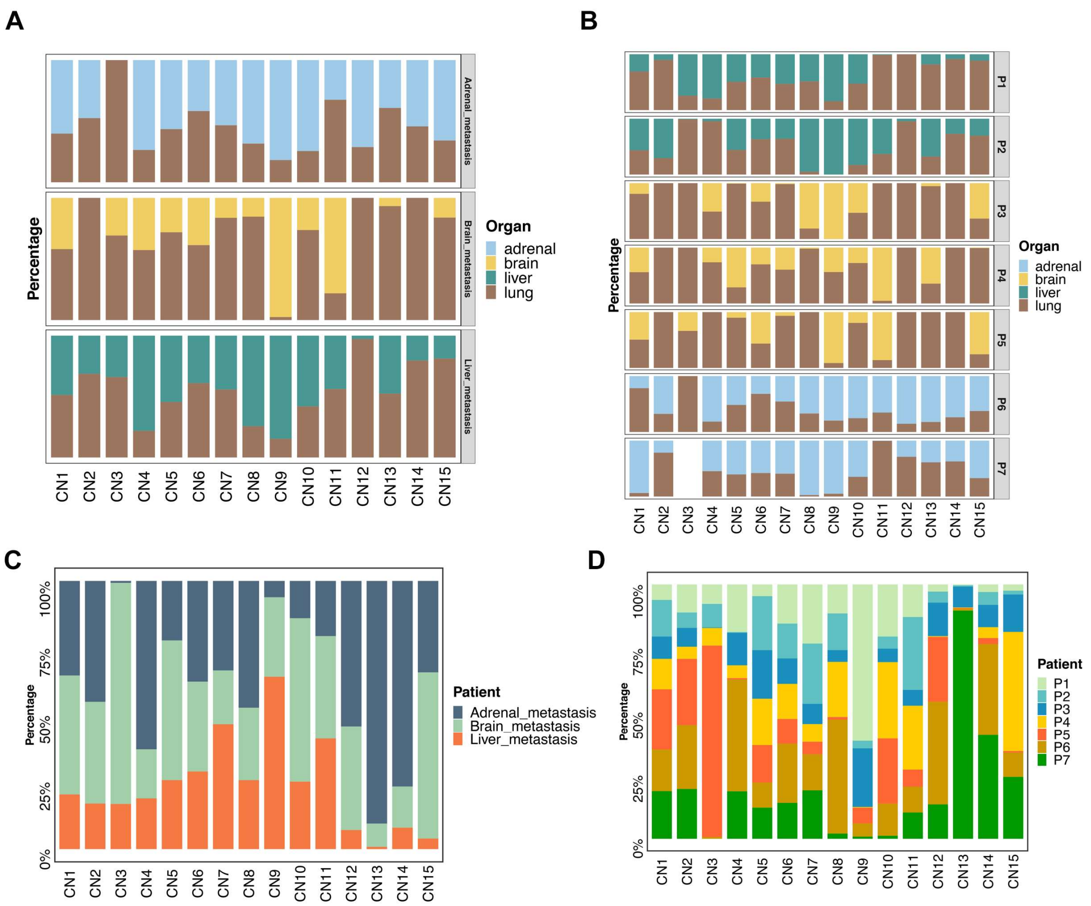

Figure S5. The frequency distribution patterns of the cell neighborhoods (CNs) on IMC images. **(A)** The distribution of each CN in paired primary and metastatic LUAD classified by the anatomical site of metastases. **(B)** The distribution of each CN in the paired primary and metastatic lesions of each patient. **(C)** The distribution of each CN in the brain, liver and adrenal gland metastases. **(D)** The distribution of each CN in each enrolled LUAD patient.

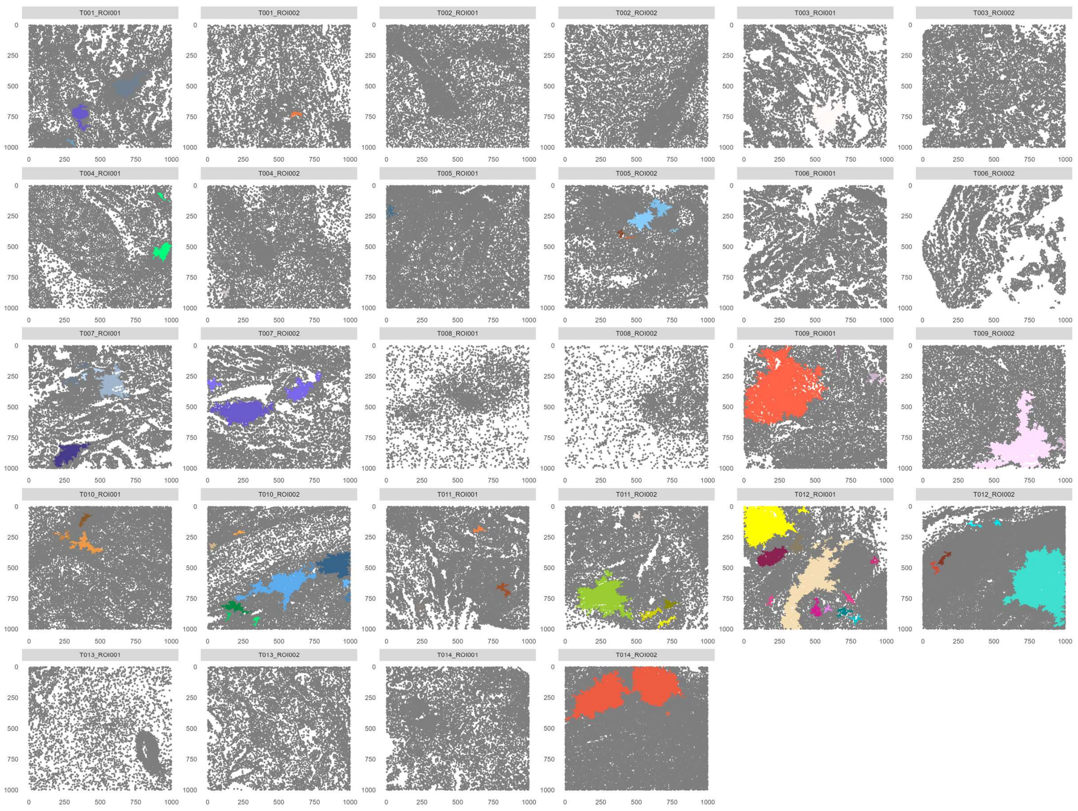

Figure S6. The Voronoi diagrams of tertiary lymphoid structure (TLS)-like structures identified by semi-supervised leaning in each sample based on the IMC.

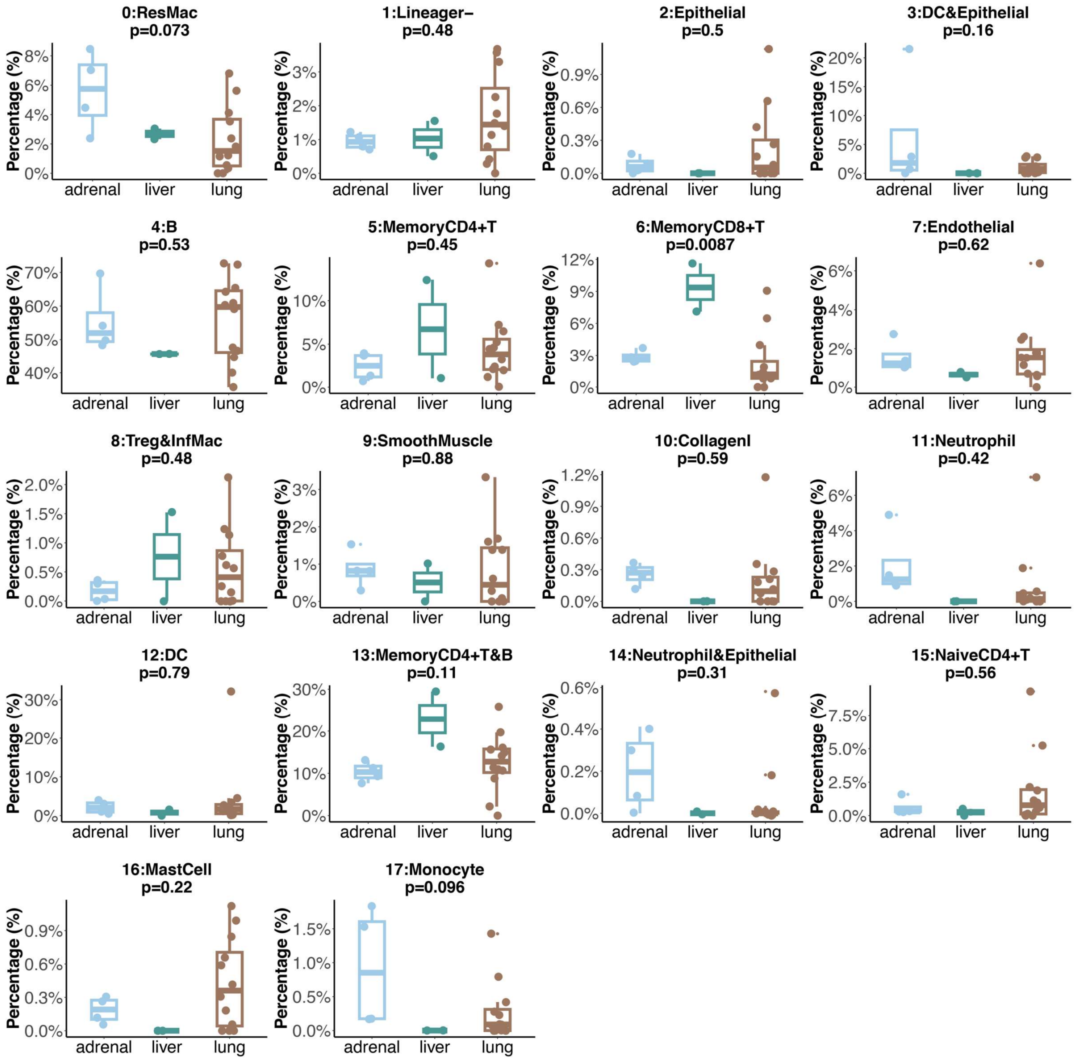

Figure S7. The distribution of each cluster in the tertiary lymphoid structure (TLS)-like structures across the primary LUAD and metastases at the liver and adrenal gland. The Wilcoxon rank-sum tests were adopted to evaluate the statistical significance.

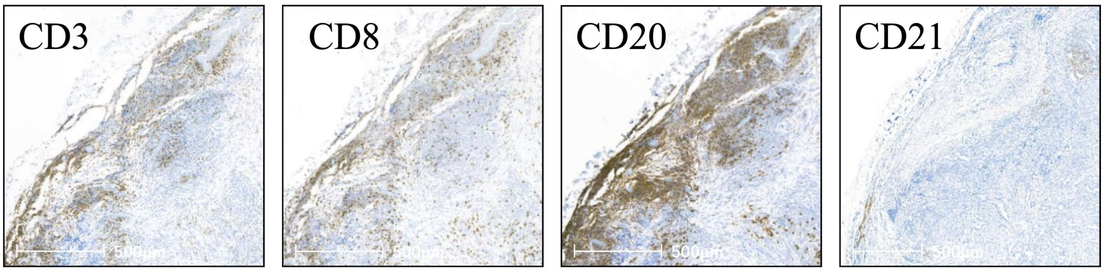

Figure S8. The presence of TLS identified by the IHC staining of CD3, CD8, CD20 and CD21.

Table S1. The key markers of each annotated cluster in IMC

**Cluster ID**	**Key markers**
Resident macrophages	$CD68^+$ $CD163^+$ $C1QC^+$
Lineage- cells	no obvious lineage marker
Epithelial cells	Pan-CK
Dendritic cells & Epithelial cells	Dendritic cells ($CD1c^+$ HLA-DR^+) & Epithelial cells (Pan-CK)
B cells	$CD20^+$
Memory $CD4^+$ T cells	$CD3^+$ $CD4^+$ $CD45RO^+$
Memory $CD8^+$ T cells	$CD3^+$ $CD8^+$ $CD45RO^+$
Endothelial cells	$CD31^+$
Treg cells & Inflammatory macrophages	Treg cells ($Foxp3^+$) & Inflammatory macrophages ($CD68^+$ $CD11b^+$ $CD15^+$ $S100A9^+$)
Smooth muscle cells	αSMA^+
Collagen I	Collagen I
Neutrophils	$CD15^+$
Dendritic cells	$CD1c^+$HLA-DR^+
Memory $CD4^+$ T & B cells	Memory $CD4^+$ T ($CD3^+$ $CD4^+$ $CD45RO^+$) & B ($CD20^+$) cells
Neutrophils & Epithelial cells	Neutrophils ($CD15^+$) & Epithelial cells (Pan-CK)
Naïve $CD4^+$ T cells	$CD3^+$ $CD4^+$ $CCR7^+$
Mast cells	$CD117^+$
Monocytes	$CD14^+$ $CD16^+$
Treg cells	$Foxp3^+$
PD-1^+ $CD4^+$ T cells	$CD3^+$ $CD4^+$ $CD45RO^+$ PD-1^+
$CD127^+$ $CD4^+$ T cells	$CD3^+$ $CD4^+$ $CD127^+$
Proliferative $CD4^+$ T cells	$CD3^+$ $CD4^+$ $CD45RO^+$ Ki-67^+
$TIM3^+$ $CD4^+$ T cells	$CD3^+$ $CD4^+$ $TIM3^+$
Cytotoxic $CD8^+$ T cells	$CD3^+$ $CD8^+$ Granzyme B^+
Memory $CD4^+$ T $cells^+$ Natural killer cells	Memory $CD4^+$ T cells ($CD3^+$ $CD4^+$ $CD45RO^+$) + NK cells ($CD57^+$)
TNF-α^+ $CD4^+$ T cells	$CD3^+$ $CD4^+$ TNF-α^+
$TIM3^+$ $CD8^+$ T cells	$CD8^+$ $TIM3^+$
$TIGIT^+$ $CD4^+$ T cells	$CD3^+$ $CD4^+$ $TIGIT^+$

Table S2. The characteristics of the LUAD patients enrolled in this study

Patient ID	Gender	Age	Primary Tumor		Metastatic Tumor			
			Histological pattern	Differentiation	Metastatic site	Metastatic pattern	Number of metastases	Time to metastases
**P1**	female	58 y	Micropapillary, Papillary, Acinar, Lepitic	Poorly differentiated	liver	metachronous	1	20.1m
**P2**	male	78 y	Solid, Micropapillary, Acinar	Poorly differentiated	liver	metachronous	1	22.5m
**P3**	female	57 y	Solid, Acinar, Papillary	Poorly differentiated	brain	metachronous	1	13.3m
**P4**	female	37 y	Micropapillary, Acinar	Poorly differentiated	brain	metachronous	1	27.4m
**P5**	male	53 y	Acinar, Lepitic	Moderately differentiated	brain	synchronous	1	-
**P6**	male	71 y	Solid	Poorly differentiated	adrenal gland	metachronous	1	4.4m
**P7**	male	65 y	Solid, Papillary, Acinar	Poorly differentiated	adrenal gland	synchronous	1	-

Table S3. Imaging mass cytometry antibody panels

Metal	Antibody	Clone	Source	Cat#	Dilution （stock solution, 0.5 mg/mL）
89Y	CD45	D9M8I	CST	47937SF	1:50
115In	S100A9	EPR3555	abcam	ab271864	1:200
141Pr	CD14	EPR3653	abcam	ab226121	1:200
142Nd	Foxp3	D2W8E	CST	74816SF	1:50
143Nd	CD16	EPR16784	abcam	ab256582	1:200
144Nd	HLA I	EPR22172	abcam	ab239788	1:400
145Nd	CD4	EPR6855	abcam	ab181724	1:100
146Nd	CD8a	C8/144B	biolegend	372902	1:100
147Sm	Collagen I	EPR7785	abcam	ab215969	1:100
148Nd	CXCL13	EPR23400-92	abcam	ab270408	1:100
149Sm	CD31	89C2	CST	85873SF	1:200
150Nd	TIM3	EPR22241	abcam	ab242080	1:50
151Eu	CD127	EPR23747-333	abcam	ab282011	1:50
152Sm	CD80	EPR1157(2)	abcam	ab271905	1:100
153Eu	TIGIT	BLR047F	abcam	ab243903	1:50
154Sm	CD163	EPR14643-36	abcam	ab215976	1:100
155Gd	IDO-1	3G2G11	proteintech	66528-1-Ig	1:200
156Gd	PD-L1	73-10	abcam	ab226766	1:50
158Gd	CCR7	EPR23192-57	abcam	ab272938	1:100
159Tb	CD68	KP1	biolegend	916104	1:400
160Gd	CD11b	EPR1344	abcam	ab209970	1:200
161Dy	CD20	IGEL/773	abcam	ab213033	1:100
162Dy	CD11c	EP1347Y	abcam	ab216655	1:100
163Dy	CD15	W6D3	biolegend	323035	1:100
164Dy	Granzyme B	EPR20129-217	abcam	ab219803	1:50
165Ho	PD-1	D4W2J	CST	63815SF	1:50
166Er	Ki-67	B56	BD	550609	1:100
167Er	CD1c	EPR23189-196	abcam	ab270797	1:100
168Er	HLA-DR	TAL 1B5	NOVUS	NB600-989	1:200
169Tm	CD117	YP145	abcam	ab216450	1:100
170Er	CD3	D7A6E	CST	24581SF	1:100
171Yb	TNFa	7B8A11	proteintech	60291-1-Ig	1:50
172Yb	IL-1b	2A1B4	proteintech	66737-1-Ig	1:50
173Yb	CD45RO	UCHL1	biolegend	304239	1:200
174Yb	CD57	NK-1	BD	555618	1:100
175Lu	C1QC	EPR2984Y	abcam	ab247391	1:100
176Yb	Pan-cytokeratin	AE-1/AE-3	biolegend	914204	1:200
194Pt	aSMA	1A4	biolegend	904601	1:100
198Pt	Vimentin	D21H3	CST	46173SF	1:200

Table S4. The corresponding tissue types of each sample in IMC

Sample ID	Patient ID	Tissue type
T001	P1	Lung
T002	P1	Liver
T003	P2	Lung
T004	P2	Liver
T005	P3	Lung
T006	P3	Brain
T007	P4	Lung
T008	P4	Brain
T009	P6	Lung
T010	P6	Adrenal gland
T011	P7	Lung
T012	P7	Adrenal gland
T013	P5	Brain
T014	P5	Lung
